# Full-Genome Characterisation of Orungo, Lebombo and Changuinola Viruses Provides Evidence for Co-Evolution of Orbiviruses with Their Arthropod Vectors

**DOI:** 10.1371/journal.pone.0086392

**Published:** 2014-01-24

**Authors:** Fauziah Mohd Jaafar, Mourad Belhouchet, Manjunatha Belaganahalli, Robert B. Tesh, Peter P. C. Mertens, Houssam Attoui

**Affiliations:** 1 Department of Vector-Borne Viral Diseases, The Pirbright Institute, Pirbright, United Kingdom; 2 Department of Pathology, University of Texas Medical Branch, Galveston, Texas, United States of America; University of Kansas Medical Center, United States of America

## Abstract

The complete genomes of Orungo virus (ORUV), Lebombo virus (LEBV) and Changuinola virus (CGLV) were sequenced, confirming that they each encode 11 distinct proteins (VP1-VP7 and NS1-NS4). Phylogenetic analyses of cell-attachment protein ‘outer-capsid protein 1′ (OC1), show that orbiviruses fall into three large groups, identified as: VP2(OC1), in which OC1 is the 2nd largest protein, including the *Culicoides* transmitted orbiviruses; VP3(OC1), which includes the mosquito transmitted orbiviruses; and VP4(OC1) which includes the tick transmitted viruses. Differences in the size of OC1 between these groups, places the T2 ‘subcore-shell protein’ as the third largest protein ‘VP3(T2)’ in the first of these groups, but the second largest protein ‘VP3(T2)’ in the other two groups. ORUV, LEBV and CGLV all group with the *Culicoides*-borne VP2(OC1)/VP3(T2) viruses. The G+C content of the ORUV, LEBV and CGLV genomes is also similar to that of the *Culicoides*-borne, rather than the mosquito-borne, or tick borne orbiviruses. These data suggest that ORUV and LEBV are *Culicoides*- rather than mosquito-borne. Multiple isolations of CGLV from sand flies suggest that they are its primary vector. OC1 of the insect-borne orbiviruses is approximately twice the size of the equivalent protein of the tick borne viruses. Together with internal sequence similarities, this suggests its origin by duplication (concatermerisation) of a smaller OC1 from an ancestral tick-borne orbivirus. Phylogenetic comparisons showing linear relationships between the dates of evolutionary-separation of their vector species, and genetic-distances between tick-, mosquito- or *Culicoides*-borne virus-groups, provide evidence for co-evolution of the orbiviruses with their arthropod vectors.

## Introduction

The genus *Orbivirus* contains 22 virus species that are formally recognised by the International Committee for the Taxonomy of Viruses (ICTV) [1], as well as multiple unclassified viruses some of which may represent additional *Orbivirus* species. The orbiviruses are vectored by *Culicoides* midges, ticks, phlebotomine flies (sandflies), and anopheline or culicine mosquitoes [1], [2]. Lebombo (LEBV) and Orungo viruses (ORUV) were originally isolated from mosquitoes [3], leading to suggestions that they might be mosquito-transmitted [1], [2].

There are four distinct ORUV serotypes (ORUV-1 to ORUV-4) that are widely distributed in tropical Africa where it has been isolated from humans, camels, cattle, goats, sheep, monkeys and *Anopheles*, *Aedes* or *Culex* mosquitoes [1], [2], [3], [4]. ORUV-1 was first isolated in Uganda during 1959 from *Anopheles funestus* mosquitoes (1 isolate) and later in Nigeria from *Aedes dentatus* (1 isolate) mosquitoes, and from humans (8 isolates) [3], [4]. Although up to 23% of the human sera tested contained neutralizing antibodies to ORUV, only a few clinical cases (involving fever, headache, myalgia, nausea, and vomiting) and three deaths were reported [5].

Transmission studies of ORUV by *Aedes* mosquitoes have been inconclusive, hampered by lack of a suitable laboratory host [6], [7] (http://wwwn.cdc.gov/arbocat). A low level of replication was detected in intra-thoracically inoculated mosquitoes, which could subsequently transmit the virus. However orally fed mosquitoes failed to replicate or transmit the virus, suggesting an insect-infection barrier. ORUV causes lethal encephalitis in suckling mice and hamsters. It also causes CPE and plaques in Vero and BHK-21 cells [8]. Mice, hamsters and chickens were not infected by subcutaneous inoculation, although mice and hamsters did produce a low-grade viraemia following intra-cranial inoculation [9].

Lebombo virus type 1 (LEBV-1 - (the only serotype of the *Lebombo virus* species) was isolated in Ibadan, Nigeria, in 1968, from a child with fever [7], [10] (http://wwwn.cdc.gov/arbocat). The virus replicates in C6/36 cells without CPE and lyses Vero and LLC-MK2 (Rhesus monkey kidney) cells. It is pathogenic for suckling mice and has also been isolated from rodents and mosquitoes (*Mansonia africana*: 1 isolate; and *Aedes circumluteolus* species) in Africa [3], [7] (http://wwwn.cdc.gov/arbocat).

The species *Changuinola virus* contains twelve ‘named’ serotypes that have been isolated from sandflies (phlebotomines) [1], [2]. Changuinola virus (CGLV) replicates in mosquito cells (C6/36) without producing CPE and is pathogenic for newborn mice or hamsters following intracerebral inoculation [11]. During a study in central Panama, seven virus strains were isolated from whole blood samples of 80 wild-caught sloths, *Bradypus variegatus* and *Choloepus hoffmanni*, using Vero cells [12]. Four strains (Pan An 59663, Pan An 53061, Pan An 307566 and Pan An 341275) were found to belong to two different serotypes and two strains belonging to the same serotype (Pan An 307566 and Pan An 341275) were associated with prolonged or recrudescent viremias in sloths. Antibodies against CGLV were widespread in both sloth species and especially prevalent in *Choloepus*, but were virtually absent from all other wild vertebrate species tested [12]. However, CGLV was also isolated in Panama from a human with a brief febrile illness, and antibodies were detected in rodents [11].

The increasing availability of representative sequence data for multiple *Orbivirus* species provides a valuable resource to study their evolution. Previous comparisons of homologous proteins of the insect and tick-borne orbiviruses, have shown only 23–38% aa identity, revealing high levels of genetic diversity within the genus [13]. We present a comparison of the genome sequences of ORUV, LEBV and CGLV, focussing on the genes coding for the viral polymerase (VP1(Pol)), the cell attachment and outer-capsid protein 1 (OC1), the sub-core shell ‘T2’ protein and the outer-core ‘T13’ protein.

## Materials and Methods

### Cell Culture and Virus Propagation

Orungo virus (UG MP 359) was isolated in 1959. Lebombo virus (SAAR 3896) was isolated in 1968. Changuinola virus (strain Xaraira, BE AR 490492) was isolated in 1990. All viruses were propagated in BHK-21 cells (clone BSR, a gift from Dr. Noel Tordo, Institut Pasteur, France), at 37°C, in Glasgow Minimum Essential Medium (GMEM) supplemented with 10% foetal bovine serum and 100 IU of penicillin/100 µg of streptomycin per ml. Infected cell cultures were incubated at 37°C for 72 hours, until cell lysis began. The cells were scraped into the supernatant and centrifuged at 3,000×g for 10 minutes. The cell pellet was used for dsRNA extraction, using RNA NOW reagent (Biogentex, Tx, USA), as described earlier [14], [15].

### Cloning of dsRNA Segments

LEBV, ORUV and CGLV genome segments were copied into cDNA, cloned and sequenced using a single primer amplification technique as previously reported [14], [15].

### Sequence Comparisons

VP1(Pol), VP2(OC1), VP3(T2) and VP7(T13) protein sequences of ORUV, LEBV and CGLV were compared with their homologues from 10 different *Orbivirus* species retrieved from international sequence databases. Sequence accession numbers used in these analyses are provided in table 1.

**Table 1:** Accession numbers of sequences downloaded from databases.

Virus or arthropod	Gene name	Accession number	Virus or arthropod	Gene name	Accession number
GIV	Seg-1	HM543465	SCRV	Seg-1	AF133431
GIV	Seg-2	HM543466	SCRV	Seg-2	AF133432
GIV	Seg-3	HM543467	SCRV	Seg-3	AF145400
GIV	Seg-4	HM543468	SCRV	Seg-4	AF145401
GIV	Seg-5	HM543469	SCRV	Seg-5	AF145402
GIV	Seg-6	HM543470	SCRV	Seg-6	AF145403
GIV	Seg-7	HM543471	SCRV	Seg-7	AF145404
GIV	Seg-8	HM543472	SCRV	Seg-8	AF145405
GIV	Seg-9	HM543473	SCRV	Seg-9	AF145406
GIV	Seg-10	HM543474	SCRV	Seg-10	AF145407
BTV-10	Seg-1	JQ740771	YUOV	Seg-1	AY701509
BTV-10	Seg-2	JQ740772	YUOV	Seg-2	AY701510
BTV-10	Seg-3	JQ740773	YUOV	Seg-3	AY701511
BTV-10	Seg-4	JQ740774	YUOV	Seg-4	AY701512
BTV-10	Seg-5	JQ740775	YUOV	Seg-5	AY701513
BTV-10	Seg-6	JQ740776	YUOV	Seg-6	AY701514
BTV-10	Seg-7	JQ740777	YUOV	Seg-7	AY701515
BTV-10	Seg-8	JQ740778	YUOV	Seg-8	AY701516
BTV-10	Seg-9	JQ740779	YUOV	Seg-9	AY701517
BTV-10	Seg-10	JQ740780	YUOV	Seg-10	AY701518
BTV-25	Seg-1	GQ982522	BTV-25	Seg-3	GQ982523
TRBV	Seg-1	HM543478	BTV-26	Seg-3	HM590643
BTV-8	Seg-1	AM498051	TRBV	Seg-2	HM543479
AHSV-2	Seg-1	FJ196584	LIPV	Seg-2	HM543476
BTV-26	Seg-1	JN255156	BTV-4	Seg-3	DQ186794
KEMV	Seg-1	HM543481	BTV-1	Seg-3	DQ186822
AHSV-1	Seg-1	AHU94887	BTV-8	Seg-3	AM498053
GIV	Seg-1	HM543465	SLOV	Seg-2	EU718677
BTV-4	Seg-1	JN255942	AHSV-4	Seg-3	AHVVP3A
EHDV-8	Seg-1	AM745057	AHSV-2	Seg-3	AM883166
LIPV	Seg-1	HM543475	BRDV	Seg-2	M87875
EHDV-1	Seg-1	AM744977	CHUV	Seg-3	AB014728
EHDV-7	Seg-1	AM745047	EHDV-8	Seg-3	AM745059
UMATV	Seg-1	HQ842619	UMAV	Seg-2	HQ842620
PHSV	Seg-1	DQ248057	EHDV-1	Seg-3	AM744979
CHUV	Seg-1	AB018086	EHDV-7	Seg-3	AM745049
BTV-2	Seg-1	JN255932	PHSV	Seg-2	DQ248058
PHSV	Seg-8	DQ248063	KEMV	Seg-2	HM543482
TRBV	Seg-8	HQ266588	MPOV	Seg-2	EF591620
KEMV	Seg-8	HQ266598	AHSV-1	Seg-2(OC1)	CAP04841
BRDV	Seg-8	M87876	AHSV-2	Seg-2(OC1)	AAN74572
CHUV	Seg-7	AB014727	AHSV-4	Seg-2(OC1)	P32553
AHSV-1	Seg-7	HM035395	BTV-8	Seg-2(OC1)	CAM57243
AHSV-4	Seg-7	D12533	BTV-1	Seg-2(OC1)	ACF37215
AHSV-2	Seg-7	FJ196591	BTV-26	Seg-2(OC1)	AED99447
EHDV-1	Seg-7	AM744983	BTV-25	Seg-2(OC1)	ACJ06702
EHDV-8	Seg-7	AM745063	BTV-4	Seg-2(OC1)	ABB71697
EHDV-7	Seg-7	AM745053	EHDV-1	Seg-2(OC1)	YP_003240109
BTV-25	Seg-7	EU839843	EHDV-8	Seg-2(OC1)	CAN89149
BTV-26	Seg-7	HM590644	EHDV-7	Seg-2(OC1)	CAN89140
BTV-4	Seg-7	EF434178	MPOV	Seg-3(OC1)	ABU95016
BTV-8	Seg-7	GQ506457	TRBV	Seg-5(OC1)	ADZ96223
BTV-1	Seg-7	GQ506450	KEMV	Seg-5(OC1)	ADZ96232
UMAV	Seg-8	HQ842626	PHSV	Seg-3(OC1)	NC_007750
UMAV	Seg-3(OC1)	AEE98370	CHUV	Seg-2(OC1)	BAD60894
*Culex pipiens*	CoxI	ADD91657	*Phlebotomus argentipes*	Antigen 5-related protein	ABA12137
*Culex caudelli*	CoxI	ADB44580	*Phlebotomus ariasi*	Antigen 5-related protein	AAX44092
*Aedes aegypti*	CoxI	AEM06325	*Lutzomia longipalpis*	Antigen 5-related protein	AF132511_1
*Aedes albopictus*	CoxI	AAX09955	*Aedes aegypti*	Antigen 5-related protein	AF466589_1
*Anopheles gambiae*	CoxI	AAR24020	*Aedes albopictus*	Antigen 5-related protein	AAV90699
*Culicoides dewulfi*	CoxI	CAJ85864	*Anopheles stephensi*	Antigen 5-related protein	AAO06821
*Culicoides scoticus*	CoxI	CAJ8581	*Anopheles darlingi*	Antigen 5-related protein	AAQ17073
*Culicoides obsoletus*	CoxI	CAJ85850	*Anopheles gambiae*	Antigen 5-related protein	AF457549_1
*Culicoides imicola*	CoxI	AAD43198	*Culicoides sonorensis*	Antigen 5-related protein	AAU06470
*Hyalomma marginatum*	CoxI	CAD24662	*Culicoides obsoletus*	Antigen 5-related protein	AGI16776
*Ixodes ricinus*	CoxI	AFV48133	*Culicoides nubeculosis*	Antigen 5-related protein	ACM40909
*Haemaphysalis longicornis*	CoxI	AFV99478	*Culex quinquefasciatus*	Antigen 5-related protein	XP_001862170
*Ixodes persulcatus*	CoxI	AEO50681	*Ixodes scapularis*	Antigen 5-related protein	XP_002410853

### Methods used for Sequence Analysis and Phylogenetic Comparisons

The genome sequences of ORUV, LEBV and CGLV were compared to available sequences for other selected reoviruses, using the DNATools package (version 5.2.018, S.W. Rasmussen: Valby Data Center, Denmark). Nucleotide (nt) and amino acid (aa) sequence alignments were generated using Clustal X version 1.8 [16]. Phylogenetic analyses were performed using MEGA5 [17]. The Neighbour-joining method [18] was used, together with a P-distance model, for initial phylogenetic reconstructions of trees. Maximum likelihood trees (nearest neighbour interchange) were then constructed using the Kimura-2 parameter model for nucleic acid sequences and Poisson model for amino acid sequences.

The best fit model of nucleotide substitution to be used in Bayesian coalescent analyses, was determined using jModelTest (v 0.1.1) [19]. Bayesian coalescent analysis based on Markov Chain Monte Carlo (MCMC) sampling [20] was implemented in BEAST (Bayesian evolutionary analysis by sampling trees) [21]. Unrooted models of phylogeny and strict molecular clock models are two extremes of a continuum [22]. Substitution rates were therefore calculated in BEAST, using a relaxed uncorrelated lognormal clock model. The most general Bayesian skyline coalescent prior was used [23], which allows for both constant and complex changes in population size through time. As a measure of estimate uncertainty, the program returns the 95% highest posterior density (HPD) interval. Molecular evolutionary rates were calculated using BEAST for the three most conserved genes that show the highest conservation in their amino acid sequences between orbiviruses: proteins VP1(Pol), T2 and T13. Although amino acids sequences are well conserved, the corresponding nucleotide sequences are more variable. Therefore to ensure a reliable alignment of the nucleotide sequences, ORFS encoding the VP1(Pol), T2 or T13 were aligned using DAMBE [24] or the web-based programme RevTrans (http://www.cbs.dtu.dk/services/RevTrans/), creating a codon to codon alignment based on the profile of amino acid alignment for corresponding proteins.

Analyses were carried out using a chain length of 10,000,000 states with the first 10% removed as burn-in. Output log files of 4 independent BEAST runs were combined together using LogCombiner (v1.5.4). This increased the effective sample sizes, and checked whether the various runs are converging on the same distribution in the MCMC run. The program Tracer (v1.5) was used to inspect posterior distributions and estimate evolutionary parameters.

The PredictProtein server (http://www.predictprotein.org) was used to predict specific localisations and interactions. Repeated aa sequences were identified using the programme REPRO (http://www.ibi.vu.nl/programs/reprowww/). The presence of nuclear localisation signals were analysed by PredictNSL, implemented in the PredictProtein server, and the cNLS Mapper (http://nls-mapper.iab.keio.ac.jp/cgi-bin/NLS_Mapper_form.cgi). Sequence relatedness to proteins in public databases was assessed using the NCBI's BLAST (http://blast.ncbi.nlm.nih.gov/Blast.cgi) and the pfam software (http://pfam.sanger.ac.uk/search/sequence).

Hydrophobicity profiles of proteins were analysed using Kyte and Doolittle algorithm [25] implemented in the Winpep programme [26].

## Results

### Sequence Analysis and Comparison of Orbivirus Proteins

The 10 dsRNA genome segments ORUV, LEBV or CGLV were converted into full-length cDNAs, cloned and sequenced. The resulting data has been deposited in GenBank (see table 2 for accession numbers). The total genome lengths of ORUV, LEBV and CGLV are 18894, 19247, 19708 nt respectively. Analyses of non-coding regions showed that genome segments of ORUV, LEBV or CGLV share conserved nucleotides at both 3′ and 5′ termini (ORUV:5′-**GUA^A^/_U_A^A^/_U_^A^/_U_---UAC**-3′, LEBV: 5′-**GUUUA^A^/_U_---^A^/_C_^A^/_C_^A^/_G_C^C^/_U_/_A_UAC**-3′, CGLV: 5′-**GUAAA^A^/_U_^A^/_U_^A^/_U_---AAACUUAC**-3′). The first three and last three nucleotides of all segments or ORUV or CGLV, and the first two and last two nucleotides of LEBV are inverted complements. In all three viruses the 5′ dinucleotide and 3′ trinucleotide are identical to those found in other orbiviruses [1], [2].

**Table 2:** Lengths of dsRNA segments, encoded putative proteins, 5' and 3' non coding regions (NCR) and G+C content of ORUV, LEBV and CGLV.

Virus/segment	Segmentlength (bp)	Protein name: length (aa)	Protein mass*(Da)	Length of 5′NCR	Conserved terminal sequences of 5′NCR	Length of 3′NCR	Conserved terminal sequences of 3′NCR	% G+C	Accession number
**ORUV**									
Seg-1	3942	VP1 : 1302	148880	12	GUAUAAU	24	UAC	44.80	JQ610675
Seg-2	2933	VP2 : 960	110863	16	GUAUAAU	37	UAC	44.60	JQ610676
Seg-3	2781	VP3 : 906	103236	17	GUAUAUA	46	UAC	45.85	JQ610677
Seg-4	1957	VP4 : 637	74148	12	GUAUAUU	34	UAC	45.84	JQ610678
Seg-5	1735	NS1 : 546	62535	35	GUAAAAA	62	UAC	45.59	JQ610679
Seg-6	1605	VP5 : 519	58106	21	GUAUAAA	27	UAC	45.73	JQ610680
Seg-7	1166	VP7 : 350	37883	16	GUAUAAA	100	UAC	50.00	JQ610681
Seg-8	1089	NS2 : 343	38534	25	GUAAAAA	35	UAC	46.83	JQ610682
Seg-9	925	VP6 : 286/NS4 : 133	31467/15592	22	GUAUAAA	45	UAC	47.24	JQ610683
Seg-10	761	NS3 : 227	25444	19	GUAUAAA	61	UAC	46.91	JQ610684
**Consensus**					**GUA^A^/_U_A^A^/_U_^A^/_U_**		**UAC**		
**LEBV**									
Seg-1	3936	VP1 : 1298	147739	18	GUUUAA	24	ACGCUUAC	46.57	JQ610665
Seg-2	3017	VP3 : 981	111818	24	GUUUAU	50	ACACCUAC	46.54	JQ610666
Seg-3	2976	VP2 : 975	112499	16	GUUUAA	35	ACACUUAC	46.44	JQ610667
Seg-4	1971	VP4 : 641	74720	8	GUUUAA	40	ACACUUAC	48.25	JQ610668
Seg-5	1736	NS1 : 549	63041	35	GUUUAA	54	ACACUUAC	47.06	JQ610669
Seg-6	1601	VP5 : 519	58795	19	GUUUAA	25	ACACUUAC	46.41	JQ610670
Seg-7	1166	VP7 : 349	37528	17	GUUUAA	102	ACACUUAC	50.34	JQ610671
Seg-8	1108	NS2 : 346	38196	21	GUUUAA	49	ACACUUAC	49.46	JQ610672
Seg-9	997	VP6 : 312/NS4 : 92	32778/11253	17	GUUUAA	44	CAACUUAC	48.24	JQ610673
Seg-10	739	NS3 : 213	23794	15	GUUUAA	85	ACGCAUAC	45.06	JQ610674
**Consensus**					**GUUUA^A^/_U_**		**^A^/_C_^A^/_C_^A^/_G_C^C^/_U_/_A_UAC**		
**CGLV**									
Seg-1	3981	VP1 : 1309	150528	12	GUAAAAUU	42	AAACUUAC	38.21	JQ610655
Seg-2	3501	VP2 : 1150	132870	16	GUAAAAUU	35	AAACUUAC	37.82	JQ610656
Seg-3	2776	VP3 : 901	103871	22	GUAAAAUU	51	AAACUUAC	41.10	JQ610657
Seg-4	1976	VP4 : 641	75661	7	GUAAAUUA	46	AAACUUAC	39.73	JQ610658
Seg-5	1908	NS1 : 563	65298	34	GUAAAUAA	86	AAACUUAC	42.62	JQ610659
Seg-6	1653	VP5 : 528	59261	29	GUAAAAUU	40	AAACUUAC	42.53	JQ610660
Seg-7	1162	VP7 : 350	38464	16	GUAAAUUA	96	AAACUUAC	44.58	JQ610661
Seg-8	1031	NS2 : 318	35330	29	GUAAAUUU	48	AAACUUAC	44.33	JQ610662
Seg-9	910	VP6 : 283/NS4 : 87	30620/11106	16	GUAAAUAA	45	AAACUUAC	45.38	JQ610663
Seg-10	810	NS3 : 233	25683	17	GUAAAAAU	94	AAACUUAC	40.86	JQ610664
**Consensus**					**GUAAA^A^/_U_^A^/_U_^A^/_U_**		**AAACUUAC**		

Most of the ORUV, LEBV and CGLV genome segments contains a single major open reading frame (ORF), which spans almost the entire length of the +ve strand. The only exceptions are Seg-9, which in each case contains two overlapping but out-of-phase ORFs. The first of which spans almost the entire length of the segment, encoding the viral helicase VP6(Hel), while a second and overlapping ORF encodes NS4, as found in other orbiviruses [27], [28]. The sizes of the encoded proteins together with the lengths of 3′ and 5′ non-coding regions (NCRs) are given for each genome segment characterised in table 2.

Comparisons of ORUV, LEBV and CGLV aa sequences (table 3) showed identity values of 10% to 68% between homologous proteins, with highest values between the T2 (67%) and T13 (68%) proteins of ORUV and LEBV. Identity levels between homologous proteins of ORUV, LEBV or CGLV, and representative insect-borne orbiviruses (BTV and YUOV) ranged from 10% to 56% (ORUV), 10% to 58% (LEBV) and 11% to 67% (GCLV) (tables 4, 5 and 6). In each case, lowest identity values were found between the highly divergent and recently identified NS4 proteins, while highest values were detected with the conserved T2 protein of BTV.

**Table 3:** Correspondence between Orungo virus (ORUV), Lebombo virus (LEBV), and Changuinola virus (CGLV).

ORUV	LEBV[%aa identity ORUV]	CGLV, [%aa identityORUV/LEBV]	Putative function*
Seg-1, VP1(Pol)	Seg-1, VP1(Pol) [60]	Seg-1, VP1(Pol) [54/56]	RNA-dependent RNA Polymerase
Seg-2, VP2(OC1)	Seg-3, VP2(OC1) [27]	Seg-2, VP2(OC1) [26/21]	Similar to outer shell protein VP2 of BTV, neutralisation epitope
Seg-3, VP3(T2)	Seg-2, VP3(T2) [67]	Seg-3, VP3(T2) [57/58]	T2, Major subcore Protein
Seg-4, VP4(Cap)	Seg-4, VP4(Cap) [53]	Seg-4, VP4(Cap) [48/49]	Minor core and capping enzyme(CaP)
Seg-5, NS1	Seg-5, NS1 [36]	Seg-5, NS1 [29/30]	Tubules (TuP)
Seg-6, VP5(OC2)	Seg-6, VP5(OC2) [59]	Seg-6, VP5(OC2) [44/44]	VP5,Outer-capsid Protein
Seg-7, VP7	Seg-7, VP7 [68]	Seg-7, VP7 [41/43]	Major core surface protein, T13 (780 copies)
Seg-8, NS2	Seg-8, NS2 [43]	Seg-8, NS2 [35/36]	Non-structural, Viral inclusion bodies (ViP)
Seg-9, VP6	Seg-9, VP6 [32]	Seg-9, VP6 [29/32]	Minor core protein, Helicase (Hel)
Seg-9, NS4	Seg-9, NS4 [13]	Seg-9, NS4 [12/10]	Non-structural
Seg-10, NS3	Seg-10, NS3 [36]	Seg-10, NS3 [28/31]	Non-structural (virus release)

* The putative functions of ORUV, LEBV and CGLV proteins by comparison to the already established functions of BTV. The functions and abbreviations (shown in parentheses) used to indicate these roles are from the *Reoviridae* chapter in the ninth taxonomy report of the ICTV. NF: Non-functional, NSI: no significant identity.

**Table 4:** Correspondence between Orungo virus (ORUV) and Great Island virus (GIV: a tick-borne orbivirus), Bluetongue virus (BTV: a typical *Culicoides*-borne orbivirus), St Croix River virus (SCRV: a tick-borne orbivirus belonging to a distinct species) and Yunnan orbivirus (YUOV) a mosquito-borne orbivirus.

ORUV	GIV, [%aa identity]	SCRV, [%aa identity]	BTV-10, [%aa identity]	YUOV-Ch, [%aa identity]
Seg-1, VP1(Pol)	Seg-1, VP1(Pol) [47]	Seg-1, VP1(Pol) [39]	Seg-1, VP1(Pol) [55]	Seg-1, VP1(Pol) [45]
Seg-2, VP2(OC1)	Seg-5, VP4(OC1) [NSI]	Seg-3, VP3(OC1) [NSI]	Seg-2, VP2(OC1) [NSI]	Seg-3, VP3(OC1) [NSI]
Seg-3, VP3(T2)	Seg-2, VP2(T2) [36]	Seg-2, VP2(T2) [23]	Seg-3, VP3(T2) [56]	Seg-2, VP2(T2) [36]
Seg-4, VP4(Cap)	Seg-3, VP3(Cap) [40]	Seg-4, VP4(Cap) [36]	Seg-4, VP4(Cap) [47]	Seg-4, VP4(Cap) [42]
Seg-5,NS1	Seg-4, NS1 [20]	Seg-6, NS1 [28]	Seg-5, NS1 [24]	Seg-5, NS1 [21]
Seg-6, VP5(OCP2)	Seg-6, VP5(OC2) [28]	Seg-5, VP5(OC2) [29]	Seg-6, VP5(OC2) [44]	Seg-6, VP5(OC2) [29]
Seg-7, VP7	Seg-7, VP7 [25]	Seg-7, VP7 [20]	Seg-7, VP7 [40]	Seg-8, VP7 [23]
Seg-8, NS2	Seg-8, NS2 [24]	Seg-8, NS2 [NSI]	Seg-8, NS2 [34]	Seg-7, NS2 [28]
Seg-9, VP6	Seg-9, VP6 [26]	Seg-9, VP6 [25]	Seg-9, VP6 [29]	Seg-9, VP6 [24]
Seg-9, NS4	Seg-9, NS4 [15]	NF ORF	Seg-9, NS4 [11]	Seg-9, NS4 [10]
Seg-10, NS3	Seg-10, NS3 [25]	Seg-10, NS3 [NS]	Seg-10, NS3 [32]	Seg-10, NS3 [25]

The functions and abbreviations (shown in parentheses) used to indicate these roles are from the *Reoviridae* chapter in the ninth taxonomy report of the ICTV [1], [2]. NF: Non-functional, NSI: no significant identity.

**Table 5:** Correspondence between Lebombo virus (LEBV) and Great Island virus (GIV: a tick-borne orbivirus), Bluetongue virus (BTV: a typical *Culicoides*-borne orbivirus), St Croix River virus (SCRV: a tick-borne orbivirus belonging to a distinct species) and Yunnan orbivirus (YUOV) a mosquito-borne orbivirus.

LEBV	GIV, [%aa identity]	SCRV, [%aa identity]	BTV-10, [%aa identity]	YUOV-Ch, [%aa identity]
Seg-1, VP1(Pol)	Seg-1, VP1(Pol) [47]	Seg-1, VP1(Pol) [38]	Seg-1, VP1(Pol) [56]	Seg-1, VP1(Pol) [45]
Seg-2, VP3(T2)	Seg-2, VP2(T2) [36]	Seg-2, VP2(T2) [23]	Seg-3, VP3(T2) [58]	Seg-2, VP2(T2) [35]
Seg-3, VP2(OC1)	Seg-5, VP4(OC1) [NSI]	Seg-3, VP3(OC1) [NSI]	Seg-2, VP2(OC1) [20]	Seg-3, VP3(OC1) [NSI]
Seg-4, VP4(Cap)	Seg-3, VP3(Cap) [40]	Seg-4, VP4(Cap) [37]	Seg-4, VP4(Cap) [50]	Seg-4, VP4(Cap) [41]
Seg-5, NS1	Seg-4, NS1 [22]	Seg-6, NS1 [21]	Seg-5, NS1 [27]	Seg-5, NS1 [24]
Seg-6, VP5(OC2)	Seg-6, VP5(OC2) [28]	Seg-5, VP5(OC2) [27]	Seg-6, VP5(OC2) [45]	Seg-6, VP5(OC2) [30]
Seg-7, VP7	Seg-7, VP7 [24]	Seg-7, VP7 [24]	Seg-7, VP7 [46]	Seg-8, VP7 [24]
Seg-8, NS2	Seg-8, NS2 [25]	Seg-8, NS2 [NSI]	Seg-8, NS2 [36]	Seg-7, NS2 [28]
Seg-9, VP6	Seg-9, VP6 [27]	Seg-9, VP6 [31]	Seg-9, VP6 [28]	Seg-9, VP6 [27]
Seg-9, NS4	Seg-9, NS4 [10]	NF ORF	Seg-9, NS4 [19]	Seg-9, NS4 [10]
Seg-10, NS3	Seg-10, NS3 [27]	Seg-10, NS3 [20]	Seg-10, NS3 [37]	Seg-10, NS3 [21]

The functions and abbreviations (shown in parentheses) used to indicate these roles are from the *Reoviridae* chapter in the ninth taxonomy report of the ICTV [1], [2]. NF: Non-functional, NSI: no significant identity.

**Table 6:** Correspondence between Changuinola virus (CGLV) and Great Island virus (GIV: a tick-borne orbivirus), Bluetongue virus (BTV: a typical *Culicoides*-borne orbivirus), St Croix River virus (SCRV: a tick-borne orbivirus belonging to a distinct species) and Yunnan orbivirus (YUOV) a mosquito-borne orbivirus.

CGLV	GIV, [%aa identity]	SCRV, [%aa identity]	BTV-10, [%aa identity]	YUOV, [%aa identity]
Seg-1, VP1(Pol)	Seg-1, VP1(Pol) [44]	Seg-1, VP1(Pol) [37]	Seg-1, VP1(Pol) [61]	Seg-1, VP1(Pol) [45]
Seg-2, VP2(OCP1)	Seg-5, VP4(OC1) [NSI]	Seg-3, VP3(OC1) [NSI]	Seg-2, VP2(OC1) [NSI]	Seg-3, VP3 [NSI]
Seg-3, VP3(T2)	Seg-2, VP2(T2) [37]	Seg-2, VP2(T2) [23]	Seg-3, VP3(T2) [67]	Seg-2, VP2(T2) [37]
Seg-4, VP4(Cap)	Seg-3, VP3(Cap) [41]	Seg-4, VP4(Cap) [38]	Seg-4, VP4(Cap) [56]	Seg-4, VP4(Cap) [40]
Seg-4, NS1	Seg-4, NS1 [26]	Seg-6, NS1 [23]	Seg-5, NS1 [31]	Seg-5, NS1 [24]
Seg-6, VP5(OCP2)	Seg-6, VP5(OC2) [31]	Seg-5, VP5(OC2) [28]	Seg-6, VP5(OC2) [51]	Seg-6, VP5(OC2) [32]
Seg-7, VP7	Seg-7, VP7 [21]	Seg-7, VP7 [24]	Seg-7, VP7 [52]	Seg-8, VP7 [22]
Seg-8, NS2	Seg-8, NS2 [25]	Seg-8, NS2 [NSI]	Seg-8, NS2 [37]	Seg-7, NS2 [33]
Seg-9, VP6	Seg-9, VP6 [26]	Seg-9, VP6 [27]	Seg-9, VP6 [39]	Seg-9, VP6 [27]
Seg-9, NS4	Seg-9, NS4 [11]	NF ORF	Seg-9, NS4 [14]	Seg-9, NS4 [11]
Seg-10, NS3	Seg-10, NS3 [25]	Seg-10, NS3 [NSI]	Seg-10, NS3 [44]	Seg-10, NS3 [25]

The functions and abbreviations (shown in parentheses) used to indicate these roles are from the *Reoviridae* chapter in the ninth taxonomy report of the ICTV [1], [2]. NF: Non-functional, NSI: no significant identity.

Comparisons to representative tick-borne orbiviruses (GIV and SCRV) showed overall identity levels of 15% to 47% (ORUV), 10% to 47% (LEBV) and 11% to 44% (CGLV) (table 3, 4 and 5), the lowest identity levels were again detected in the NS4 proteins, with highest values in the highly conserved polymerase (VP1) of GIV (44 to 47%). In contrast to the insect borne viruses (BTV and YUOV), aa identities between the T2 proteins of ORUV, CGLV or LEBV and those of GIV or SCRV were considered to be below significant levels (<10%) (table 3, 4 and 5).

The NS4 sequences of ORUV, LEBV and CGLV contain a high proportion of charged residues, with basic R+K (arginine+lysine) content ranging from 13% to 22%, while acidic E+D (glutamic+aspartic acids) content ranges from 12% to 22%. Each NS4 protein contains 4–5 histidine residues. As seen in other orbivirus NS4s [27], these analyses also identified either mono-partite or bi-partite nuclear localisation signals (NLS) (table 7). The 3 NS4s are rich in arginine and lysine residues that are essential for NLS [29]. The NS4 of ORUV (133 aa long), LEBV (92 aa long) and CGLV (87 aa long) were also predicted, using BLAST and Pfam analyses, to bind DNA, confirming previous results obtained with NS4s of GIV and BTV [27] and in particular the ORUV NS4 exhibited 30% amino acid identity with the XRE transcriptional regulation factor (binds DNA and regulates transcription). These findings confirm the presence of NS4 ORF in sandfly-borne orbiviruses as recently shown in other insect- and tick-borne orbiviruses [27].

**Table 7:** Sequences of ORUV, LEBV or CGLV NLS’s.

Virus	NLS monopartite	NLS Bipartite	positions
PHSV	RKLERVEMERKMKK		86–99
PHSV	RKMKKSEVNKARRKL		95–109
YUOV	RTPERVESVKKRLN		99–112
EHDV	RHRKGAKRKR		43–13
BTV	RKRAAKRLKMQMW		12–24
AHSV	RRTRVKRKRTKY		4–15
AHSV	RTRVKRKRTKY		5–15
AHSV	RVKRKRTKYM		7l–16
GIV		RKRGLEFLLLPLHEYVTHCAKEDIRIYES	113–141
CGLV	KKQKRRIRR		25–33
CGLV	QKRRIRR		27–33
CGLV	KRRIRREKIKTEREVTRKRR		28–47
CGLV	TRKRRQ		43–48
LEBV	LERKRRGWRV		77–86
LEBV		RIRVGNIKQAEEQLLGMRDRLEDALERKRRGW	53–84
ORUV	KRRRL		36–40
ORUV	RRRLEEVRIQSSGKVEMEGDKLRRLK		37–62

Comparison to NLSs of other insect-borne and tick-borne orbiviruses.

### Comparisons of the VP1(Pol) to the Polymerase of other Orbiviruses

**Figure 1:**
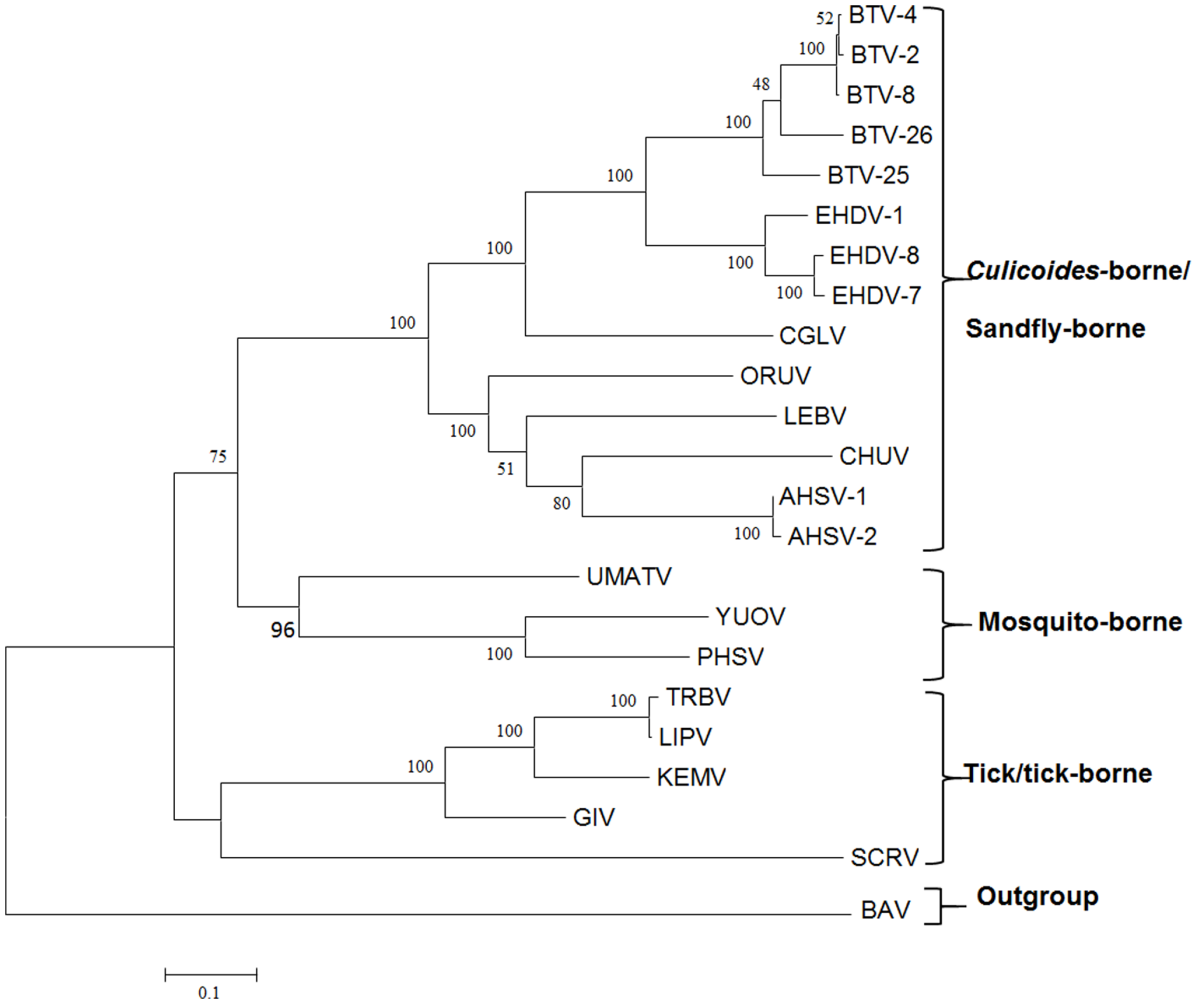
Maximum likelihood trees showing phylogenetic comparisons of the aa sequences of VP1 of ORUV, LEBV and CGLV, aligned with those of other *Orbivirus* species. Figure 1 is an ML amino acid tree, respectively, both depicting the three groups of orbiviruses (i-*Culicoides*−/sandfly-borne, ii- mosquito-borne and iii- tick-borne) as separate clusters. The polymerase of Banna virus (genus *Seadornavirus*, family *Reoviridae*: a 12-segmented mosquito-borne dsRNA virus) used as outgroup. This figure shows the root to be located between the tick/tick-borne orbiviruses and the insect-borne orbiviruses. LEBV, ORUV and CGLV all cluster among *Culicoides*-borne orbiviruses. The scale bar represents the number of substitutions per site.

Phylogenetic comparisons of the polymerase genes and proteins of ORUV, LEBV and CGLV were aligned with those of other *Orbivirus* species (figure 1 and figure S1), showing that the tick and tick-borne viruses cluster together, ‘rooting’ the insect-borne orbiviruses. A previous study detected 53% to 73% identity in VP1(Pol) between different insect transmitted *Orbivirus* species, including AHSV, EHDV, BTV, *Equine encephalosis virus* (EEV) and *Palyam virus* (PALV) [13]. In contrast only ∼35% aa identity was detected between these insect transmitted viruses, and the tick-orbivirus SCRV; and 45% between the insect transmitted viruses and members of the tick-borne *Great Island virus* species (GIV). Intermediate identity levels of 41% were detected between the polymerases of GIV and SCRV [30]. Accession numbers for orbivirus VP1(Pol) downloaded from the databases are provided in table 1.

Comparisons of VP1(Pol) of ORUV with the *Culicoides*-borne orbiviruses, showed 50% to 62% aa identity with Ibaraki virus (EHDV-2) and AHSV, respectively. In contrast, comparison of ORUV VP1 with the mosquito-borne orbiviruses showed 47% to 49% aa identity with PHSV and Umatilla virus (UMAV), respectively. Amino acid identities with tick-borne orbivirus VP1 ranged from 39% to 47% with SCRV and GIV, respectively.

Comparisons of VP1(Pol) of LEBV with the *Culicoides*-borne orbiviruses, showed 51% to 62% aa identity with EHDV-2 and AHSV, respectively. In contrast, comparison of LEBV VP1 with the mosquito-borne orbiviruses showed 47% to 49% aa identity with PHSV and UMAV, respectively. Amino acid identities with tick-borne orbivirus VP1 ranged from 38% to 47% with SCRV and GIV, respectively.

Comparisons of VP1(Pol) of CGLV with the *Culicoides*-borne orbiviruses, showed 52% to 61% aa identity with Equine encephalosis virus (EEV) and BTV, respectively. In contrast, comparison of CGLV VP1 with the mosquito-borne orbiviruses showed 45% to 48% aa identity with PHSV and UMAV, respectively. Amino acid identities with tick-borne orbivirus VP1 ranged from 38% to 47% with SCRV and GIV, respectively.

Amino acid identity levels between ORUV, LEBV and CGLV, in the VP1(Pol) ranged from 54% to 60% (table 6).

### Comparisons of the T2 Subcore Proteins

The orbiviruses show 26% to 83% aa identity in their T2 proteins between different virus species [13]. The levels of aa identity between the T2 proteins of ORUV, LEBV and CGLV ranged from 57% to 67% (table 6) confirming their classification as three different species.

**Figure 2:**
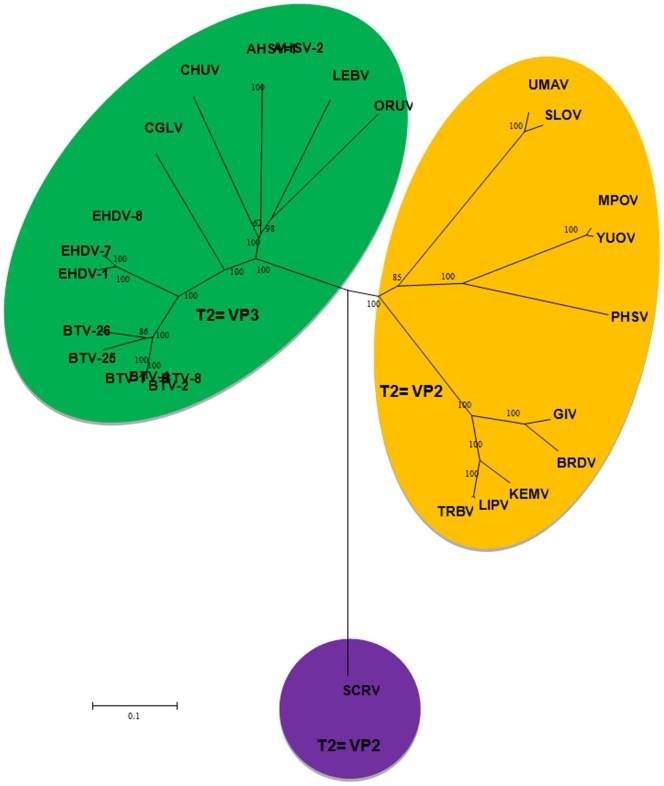
A neighbour joining tree showing phylogenetic comparisons the aa sequences of T2 (VP2(T2) of mosquito-borne and tick-borne orbiviruses and VP3(T2) of *Culicoides*-borne orbiviruses) aligned with those of ORUV, LEBV and CGLV. The tree depicts the groups of *Culicoides*-borne and sandfly-borne viruses having their VP3 as the T2 protein, while the tick-borne and mosquito-borne viruses having their VP2 as the T2 protein. LEBV, ORUV and CGLV all cluster among *Culicoides*-borne orbiviruses. The scale bar represents the number of substitutions per site.

The sub-core-shell proteins of ORUV, LEBV and CGLV were identified as (VP3)T2, the third largest viral-protein in each case, by phylogenetic comparisons to VP3(T2) of BTV [31] and VP2(T2) of GIV [13], [30]. The aa/nt trees for the T2 proteins/genes, have a similar topology, showing that ORUV (VP3), LEBV (VP3) and CGLV (VP3) cluster together as related but distinct virus species within the ‘VP3(T2)/*Culicoides*-borne group’ (Figure 2). In contrast the mosquito-borne orbiviruses cluster together as a ‘VP2(T2)’ group. This clustering contradicts previous suggestions that ORUV and LEBV are mosquito-borne viruses [3], [5], [8], [10].

Accession numbers for orbivirus T2 proteins downloaded from the databases are provided in table 1.

The aa sequence of VP3(T2) from ORUV exhibit 55% (BTV) to 63% (Palyam virus, PALV) identity to the other *Culicoides*-borne orbiviruses; 35% (PHSV) to 36% (YUOV and UMAV) identity to the mosquito-borne orbiviruses; and only 23% (SCRV) to 36% (GIV) with tick-borne orbiviruses. The VP3(T2) of LEBV shows 59% (BTV and EHDV) to 67% (AHSV) aa identity to the T2 of the *Culicoides*-borne orbiviruses, 35% (PHSV and YUOV) to 36% (UMAV) identity to mosquito-borne orbiviruses and only 23% (SCRV) to 36% (GIV) to the tick-borne orbiviruses. VP3(T2) of CGLV shows 52% (EEV) to 71% (Tilligery virus, TILV and EHDV) aa identity to the *Culicoides*-borne orbiviruses, 36% (UMAV) to 37% (PHSV and YUOV) to the mosquito-borne orbiviruses and only 37% (SCRV) to 38% (GIV) with the tick-borne orbiviruses.

### Comparisons of VP1(Pol), T2 and T13 Amino Acid Trees, to Trees of Cytochrome Oxidase I or Antigen 5–related Proteins of Arthropod Vectors

We have used sequence comparisons and trees to compare the ancestry and evolution of cytochrome oxidase I (COXI) and antigen 5-related proteins (which are both available from sequence databases for various arthropods), to those of the three most conserved orbivirus genes: VP1(Pol), T2 and VP7(T13).

**Figure 3:**
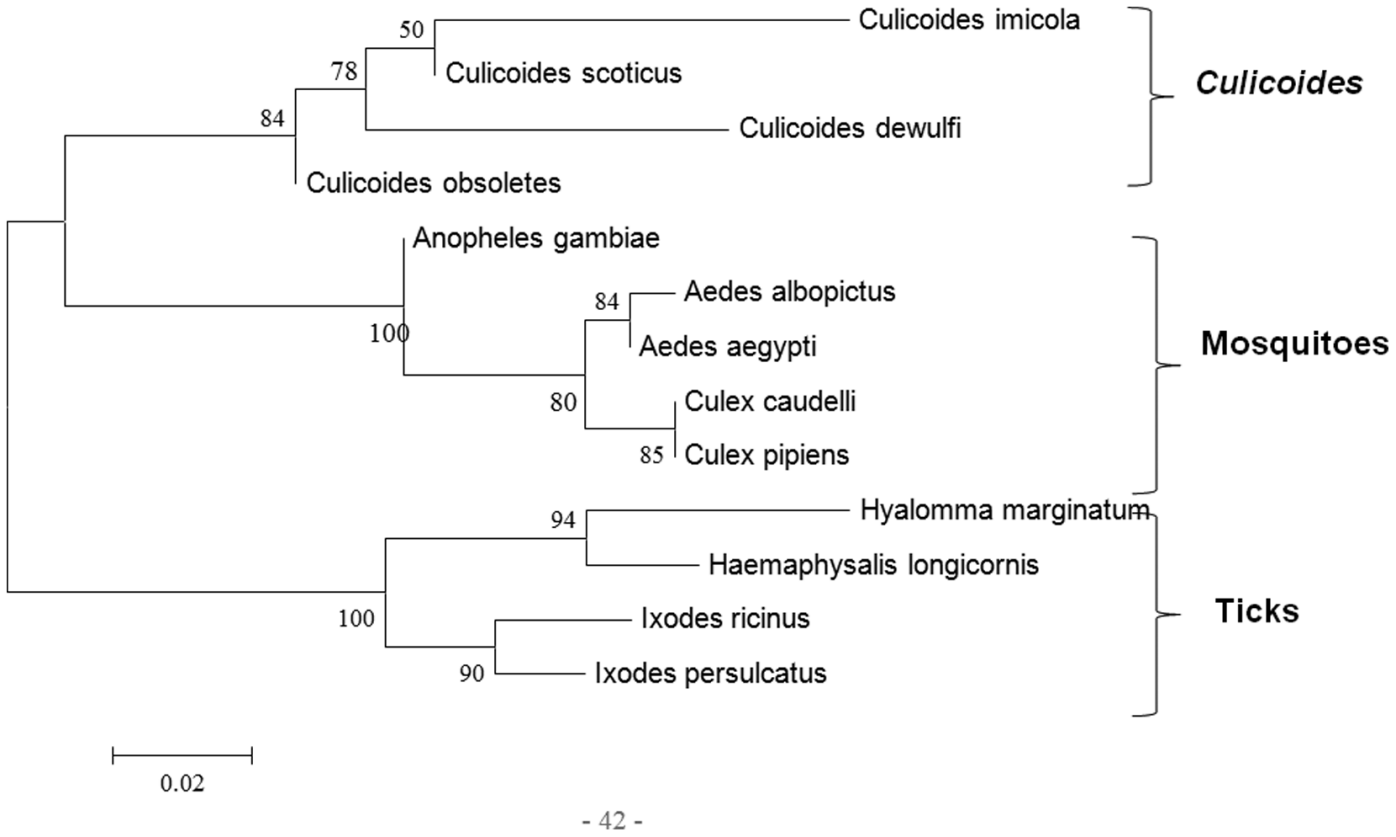
Maximum likelihood trees showing phylogenetic comparisons of the amino acid of cytochrome oxidase I (COXI) of arthropods. ML tree of COXI of 3 groups of arthropods which transmit orbiviruses (ticks, mosquitoes and *Culicoids*). The scale bar represents the number of substitutions per site.

**Figure 4:**
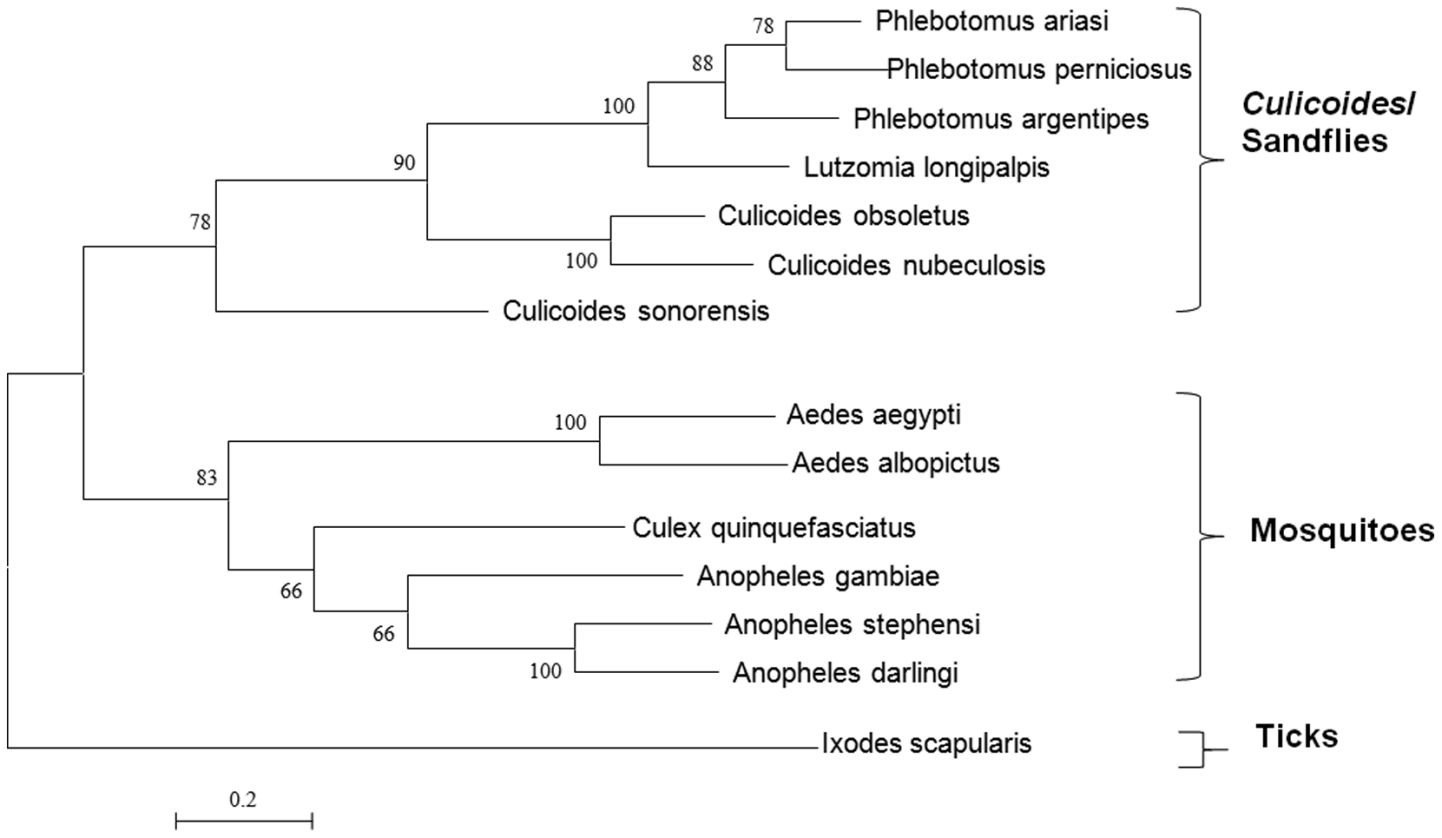
Maximum likelihood trees showing phylogenetic comparisons of the amino acid of antigen 5-related proteins of arthropods. ML tree of the antigen 5-related proteins of all 4 groups of arthropods (ticks, mosquitoes, *Culicoids* and sandflies) depicting *Culicoides* and sandflies as one cluster. The scale bar represents the number of substitutions per site.

Previous evolutionary analyses have suggested that ticks appeared approximately 225 million years ago (MYA) [32], whilst the earliest dating of culicine mosquitoes is about 150 MYA [33] and *Culicoides* biting midges have been dated to the Cretaceous period (140-65 MYA) [34], [35]. The earliest extant lineage of biting midges was found in 120–122 million years old amber [36]. The oldest sandflies were identified in Lebanese amber that is 135–120 million years old [37], [38]. The evolutionary and fossil studies are in agreement regarding dates of separation of ticks, mosquitoes and *Culicoides*. They however disagree on the date of separation of sandflies [39]. The use of two different arthropod genes to assess arthropod phylogenies was therefore important. The COXI based tree for 3 groups which transmit orbiviruses (ticks, mosquitoes and *Culicoides*) is shown in figure 3. The antigen 5-related protein based tree of all 4 arthropod groups is shown in figure 4.

**Figure 5:**
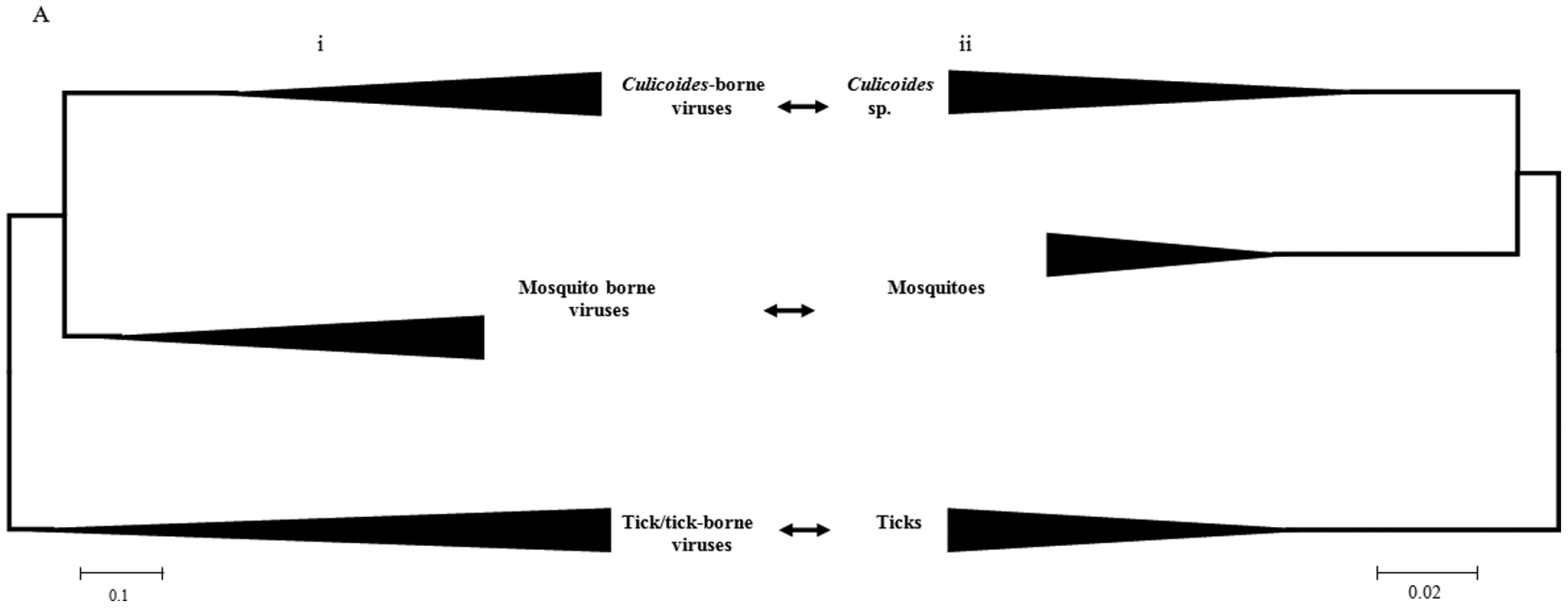
Comparison of topologies of orbivirus VP1(Pol) tree in (i) to vector COXI proteins in (ii). Comparison of topologies of orbivirus VP1(Pol) tree in (i) to that of vector COXI (*Culicoides*, mosquitoes and ticks) in (ii). The topologies of the vector proteins based trees mirror those of the VP1(Pol) trees of orbiviruses. The scale bar represents the number of substitutions per site.

**Figure 6:**
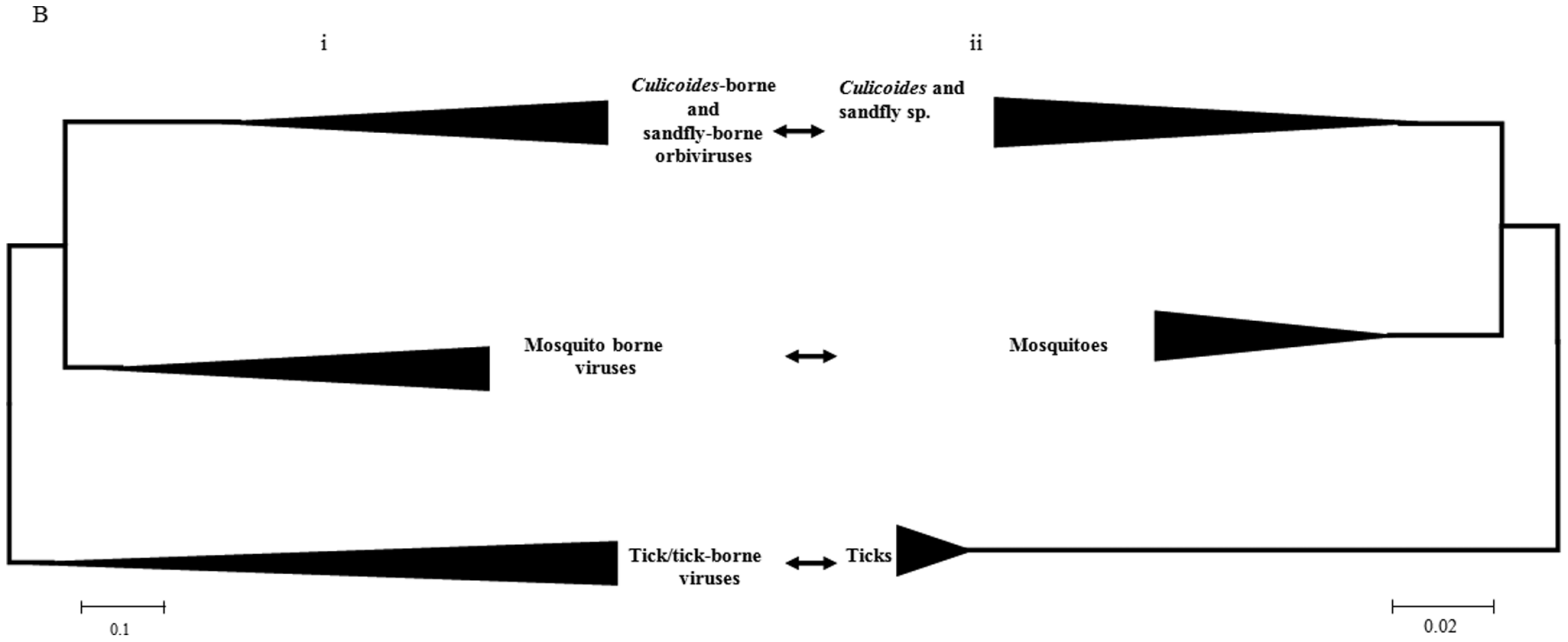
Comparison of topologies of orbivirus VP1(Pol) tree in (i) to vector antigen 5-related proteins in (ii). Comparison of topologies of orbivirus VP1(Pol) tree in (i) to that of vector antigen 5-related proteins (*Culicoides*, sandflies, mosquitoes and ticks) in (ii). The topologies of the vector proteins based trees mirror those of the VP1(Pol) trees of orbiviruses. The scale bar represents the number of substitutions per site.

Comparisons of the VP1 trees, to COXI tree of ticks, mosquitoes and *Culicoides* also revealed strikingly similar topologies (figure 5). The antigen 5-related protein based tree showed an identical topology to that of the VP1 trees (figure 6). Such a resemblance has been considered as an indication of co-evolution of viruses and their hosts [40].

Topologies of trees for the T2 aa and nt sequences differed from the VP1 trees. The orbivirus T2 protein sequences cluster into two groups: containing either the VP2(T2) tick-borne viruses, or the VP3(T3) mosquito-borne/*Culicoides*-borne viruses. This clustering indicates that the mosquito-borne T2 sequences are closer to those of the tick-borne, than the *Culicoides*-borne viruses.

### Comparisons of the T13 (VP7) Core Surface Proteins

Accession numbers for orbivirus VP7(T13) downloaded from the databases are provided in table 1.

Sequence analyses show that VP7(T13) of ORUV exhibits 40% (BTV) to 50% (AHSV) aa identity to other *Culicoides*-borne orbiviruses, only 20% (UMAV) to 23% (PHSV and YUOV) aa identity to the VP7(T13) of mosquito-borne orbiviruses and 20% (SCRV) to 25% (GIV) with sequenced tick-borne orbiviruses. VP7(T13) of LEBV shows only 41% (Eubenangee virus, EUBV) to 51% (AHSV) aa identity to the VP7(T13)of all sequenced *Culicoides*-borne orbiviruses, 22% (UMAV) to 24% (PHSV and YUOV) aa identity to the VP7(T13) of all sequenced mosquito-borne orbiviruses and only 24% (SCRV or GIV) with sequenced tick-borne orbiviruses. The VP7(T13) of CGLV shows only 42% (EEV and PALV) to 58% (EHDV) aa identity to the VP7(T13)of all sequenced *Culicoides*-borne orbiviruses, 20% (PHSV) to 23% (UMAV) aa identity to the VP7(T13) of all sequenced mosquito-borne orbiviruses and only 18% (SCRV) to 21% (GIV) with sequenced tick-borne orbiviruses.

**Figure 7:**
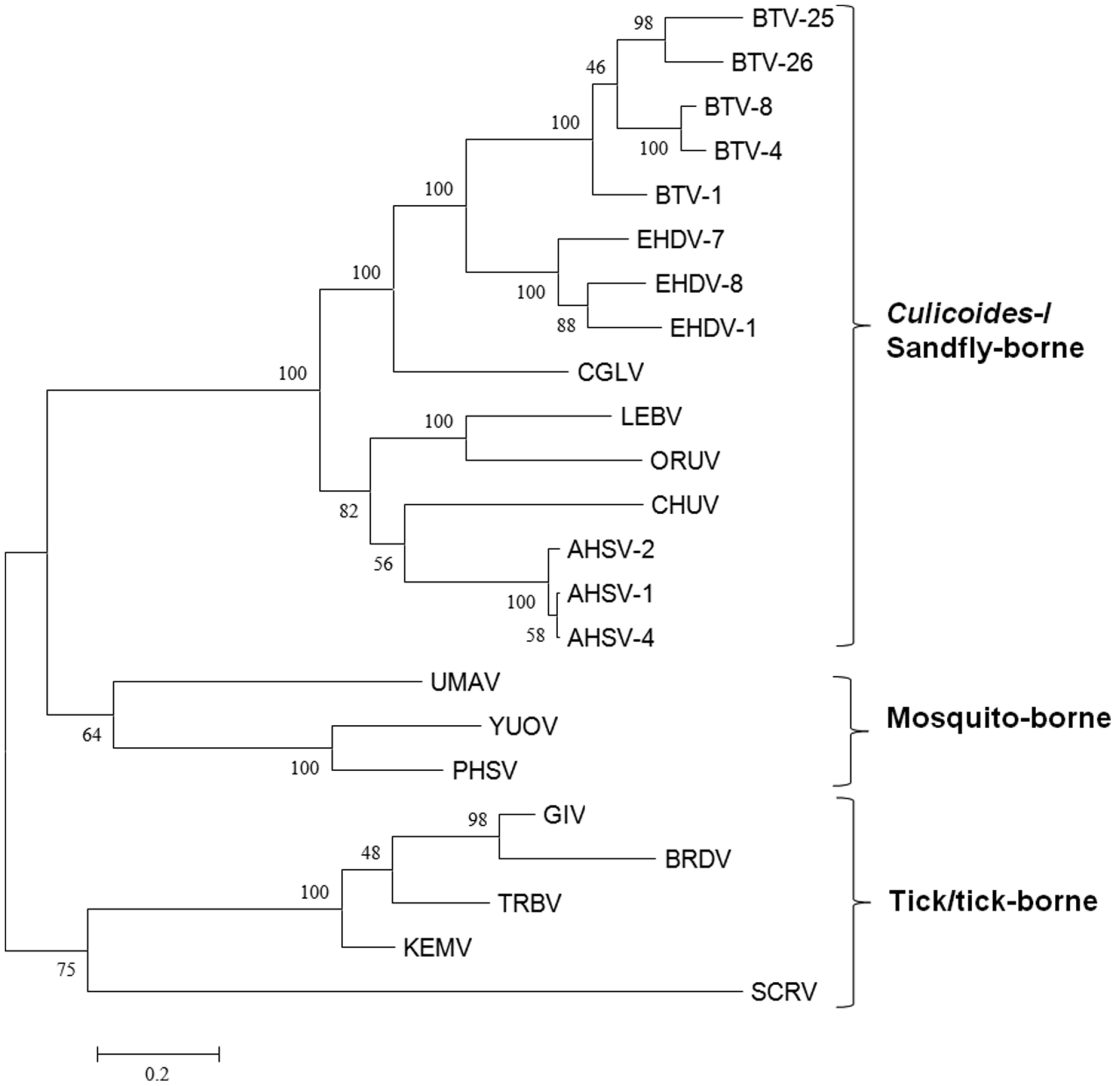
Maximum likelihood trees showing phylogenetic comparisons of the nucleotide sequences of the genome segment encoding the VP7(T13) proteins of ORUV, LEBV and CGLV, aligned with those of other *Orbivirus* species. The nucleic acid sequences were aligned based on the profile of aa alignments generating a codon to codon alignment, showing the three groups of orbiviruses (i-*Culicoides*−/sandfly-borne, ii- mosquito-borne and iii- tick-borne) as separate clusters. LEBV, ORUV and CGLV all cluster among *Culicoides*-borne orbiviruses. The scale bar represents the number of substitutions per site.

Accordingly, VP7(T13) of ORUV, LEBV and CGLV shows highest aa identity levels compared to other *Culicoides*-borne orbiviruses, but is less closely related to the mosquito-borne and tick-borne orbiviruses. The aa maximum likelihood phylogenetic tree (figure S2) confirms that VP7(T13) of ORUV, LEBV and CGLV clusters within the *Culicoides*-borne virus-group. A codon to codon aligned nucleic acid ML tree (figure 7) showed a similar topology to those of VP1(Pol), where tick-borne viruses provide a root to insect-borne orbiviruses. The VP7 nucleic acid ML tree has a strikingly similar topology to that of the arthropod COXI-based and antigen 5-related-based protein trees, consistent with the ‘co-evolution’ hypothesis.

### Comparison of the Genetic Distances between Groups of Orbiviruses and Dates of Vector-family Divergence

The largest genetic distance between members of the tick-borne, mosquito-borne or *Culicoides*-borne groups, were calculated for VP1(Pol), T2 (VP2 or VP3) and the VP7(T13) (the three most conserved orbivirus proteins) (table 8).

**Table 8:** Genetic distances between the most divergent viruses among tick-borne, mosquito-borne or *Culicoides*-borne orbiviruses.

Protein	Tick-borne	Mosquito-borne	*Culicoides*-borne
**VP1**	78	51	44
**T2**	68	53	46
**T13**	82	70	57

**Figure 8:**
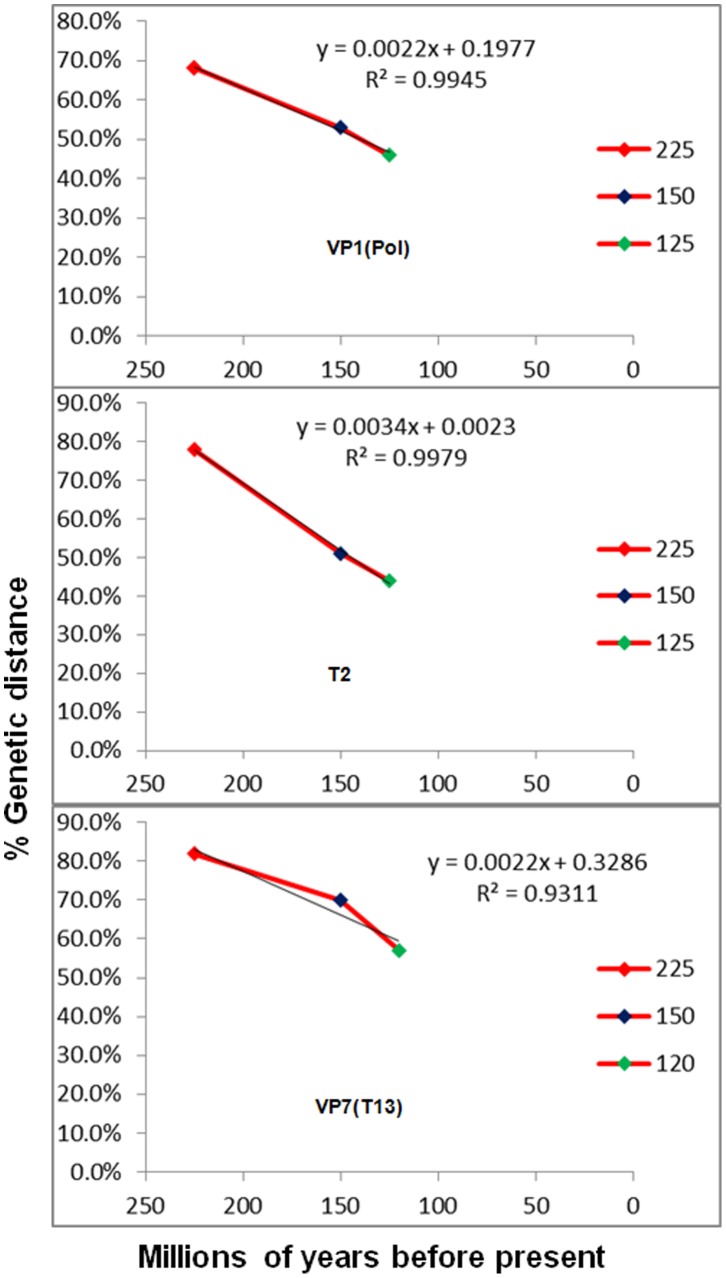
Linear relationships linking the largest genetic distances between tick-borne, mosquito-borne or *Culicoides*-borne orbiviruses and time of divergence for vectors. The largest genetic distance within a group of orbiviruses (tick-borne, mosquito-borne or *Culicoides*-borne) is plotted against the date of separation of vector groups (ticks, mosquitoes or midges). A linear relationship is depicted for both the VP1(Pol) and the T2 (correlation coefficient R^2^>0.99), and is less obvious in the T13 protein (R^2^ = 0.9311).

Comparisons of the divergence dates for the ticks, mosquitoes and *Culicoides* midges, with the genetic distances between the orbiviruses transmitted by these three vector groups showed nearly linear relationships for both VP1 and T2 proteins, with correlation coefficients of 0.998 and 0.994, respectively. The linearity in the T13 protein is less obvious, with a correlation coefficient of 0.931 for that series (figure 8).

### G+C Content of the Orbivirus Genome

Analysis of the G+C contents of genomes of various midge-borne, mosquito-borne and tick-borne orbiviruses showed specificities to each group. The G+C content of *Culicoides*-borne viruses ranged from 42 to 44% (examples: 44.10% for BTV, 42.14% EHDV, 42.68%AHSV) (table 2). The G+C content of mosquito-borne orbiviruses ranged from 35 to 41% (examples 40.4% for YUOV, 35.7% for PHSV, 40.94% for UMAV, 40.1% for CHUV). For tick-borne orbiviruses the G+C content ranged from 52 to 58% (examples: 58.2 for GIV, 57.9% for BRDV, 52% SCRV) (table 2).

The G+C content of the three viruses sequences reported in this paper are 46.3% for ORUV, 47.4% for LEBV and 41.7% for CGLV similar to those of other midge-borne viruses. These results support phylogenetic clustering of virus genes of ORUV, LEBV and CGLV within the VP3(T2) group, containing principally the midge-borne viruses (table 2).

### Calculations of Molecular Evolutionary Rates (MRE)

Molecular evolutionary rates (MREs) were calculated for the three most conserved orbivirus genes using BEAST and were consistent with what is known for RNA viruses in general. The overall mean rates were 3.22×10^−4^ (95% HPD = 2.18×10^−4^ to 3.99×10^−4^) for VP1(Pol) gene, 1.58×10^−4^ (95% HPD = 1.11×10^−4^ to 2.77×10^−4^) for the T2 gene and 4.13×10^−4^ (95% HPD = 3.55×10^−4^ to 4.89×10^−4^) for the VP7 gene. Previous studies have indicated higher evolutionary rates for the insect-borne flaviviruses viruses (1.62×10^−4^–8.54×10^−4^), as compared 1.22×10^−4^–7.28×10^−5^ for the tick-borne arboviruses [41].

The MREs calculated for the insect-borne orbiviruses are 3.51×10^−4^ (95% HPD = 2.41×10^−4^ to 4.15×10^−4^) for the VP1(Pol) gene, 2.3×10^−4^ (95% HPD = 1.73×10^−4^ to 3.16×10^−4^) for the T2 gene and 4.52×10^−4^ (95% HPD = 3.83×10^−4^ to 5.35×10^−4^) for the VP7 gene. In contrast lower MREs were calculated for the tick-borne orbiviruses were 1.91×10^−4^ (95% HPD = 0.72×10^−4^ to 2.87×10^−4^) for VP1(Pol), 0.96×10^−4^ (95% HPD = 0.75×10^−4^ to 1.46×10^−4^) for the T2 protein and 2.43×10^−4^ (95% HPD = 1.83×10^−4^ to 3.38×10^−4^) for the VP7 gene.

MREs for the insect-borne orbiviruses are almost double those of the insect-borne orbiviruses.

### Outer Capsid Protein 1 of the Insect-borne Orbiviruses Represents a Concatemer of an Ancestral Tick-borne Counterpart

**Figure 9:**
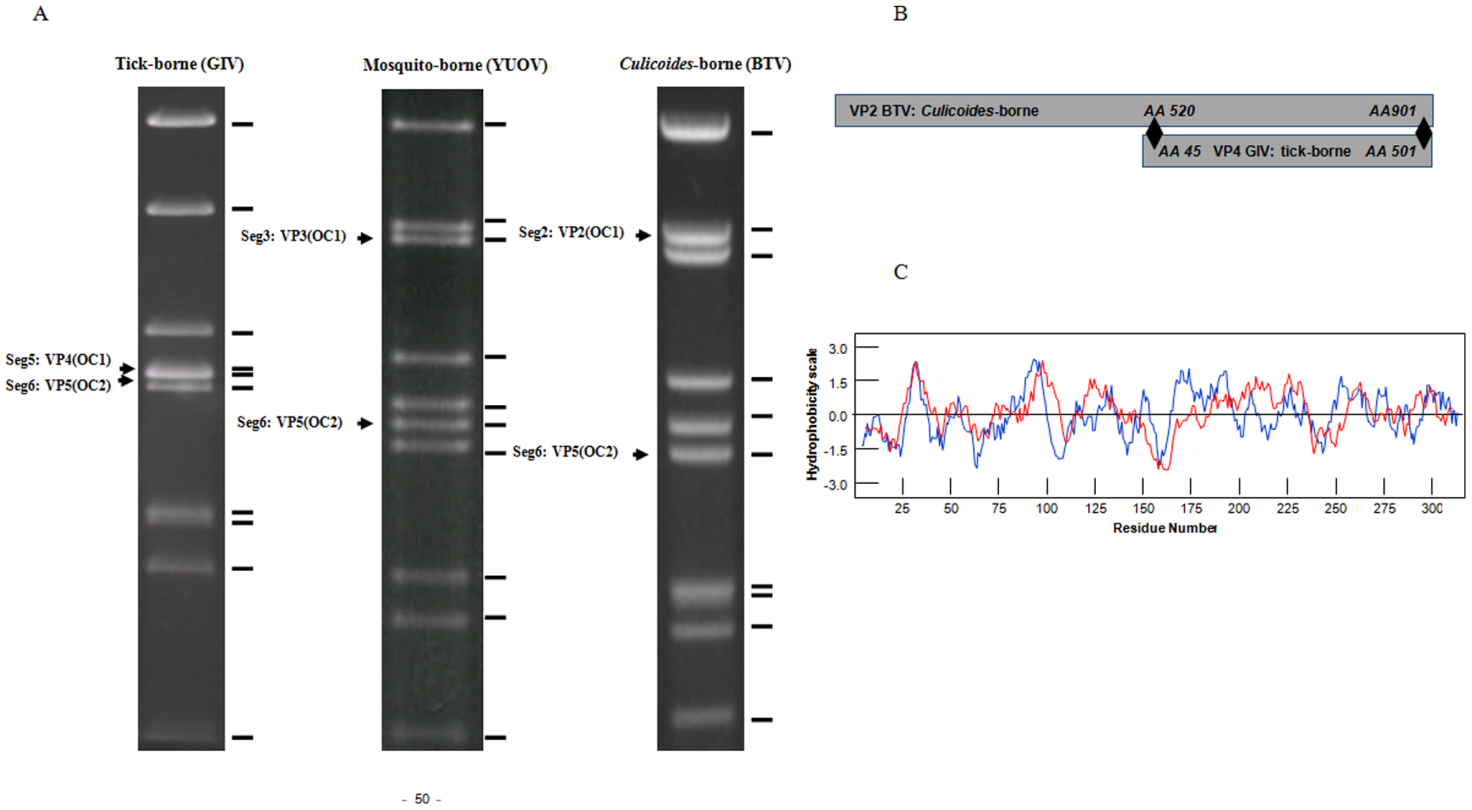
Electropherotypes of a tick-borne (GIV), mosquito-borne (YOUV) and *Culicoides*-borne (BTV) orbivirus and relatedness of the GIV VP4 to BTV VP2. A: Electropherotypes of Great Island virus (GIV), Yunnan orbivirus (YUOV) and Bluetongue virus (BTV) showing the genome segments encoding OC1 and OC2 in tick-, mosquito and *Culicoides*-borne orbiviruses. **B:** a schematic of the match between the VP4(OC1) of GIV and VP2(OC1) of BTV. VP4 (OC1) of tick-borne orbiviruses is 55% the length of VP2(OC1) of *Culicoides*-borne or VP3(OC1) of mosquito-borne orbiviruses. Amino acids 45–501 of VP4 matches the COOH terminal half of OC1s of insect-borne orbiviruses (e.g: VP2(OC1) of BTV: aa 520–901). **C:** Hydrophobicity profiles of GIV VP4 and domain 2 of BTV VP2 (VP2D2); the two profiles are broadly similar and show the plot of aa 114 to 523 of VP4 (blue line) superimposed onto that of aa 642 to 956 of VP2D2 (red line).

Agarose gel electropherotypes of a *Culicoides*-borne orbivirus (BTV), mosquito-borne orbivirus (YUOV) and a tick-borne orbivirus (GIV) are shown in figure 9. These electropherotypes show the relative mobility (related to the size) of genome segments encoding OC1 and OC2 of these groups of orbiviruses. The relative migration of genome segments encoding OC1s indicate that Seg-5 encoding VP4(OC1) of the tick-borne viruses is about half the size of that encoding VP2(OC1) of *Culicoides*-borne and VP3(OC1) of mosquito-borne viruses. VP4 of GIV is related to the carboxy terminal half (aa 483 to 954) of VP2 from BTV, EHDV, ASHV (*Culicoides* transmitted) and VP3 from YUOV and PHSV (both mosquito-transmitted), with 28–30% aa sequence identity. Figure 9 also shows a schematic for the match between VP4(OC1) of GIV and the carboxy terminal half of VP2(OC1) of BTV. The hydrophobicity plot of GIV VP4 between aa 114 to 523 is similar to that of aa 642 to 956 of VP2 (VP2D2) (figure 9).

**Figure 10:**
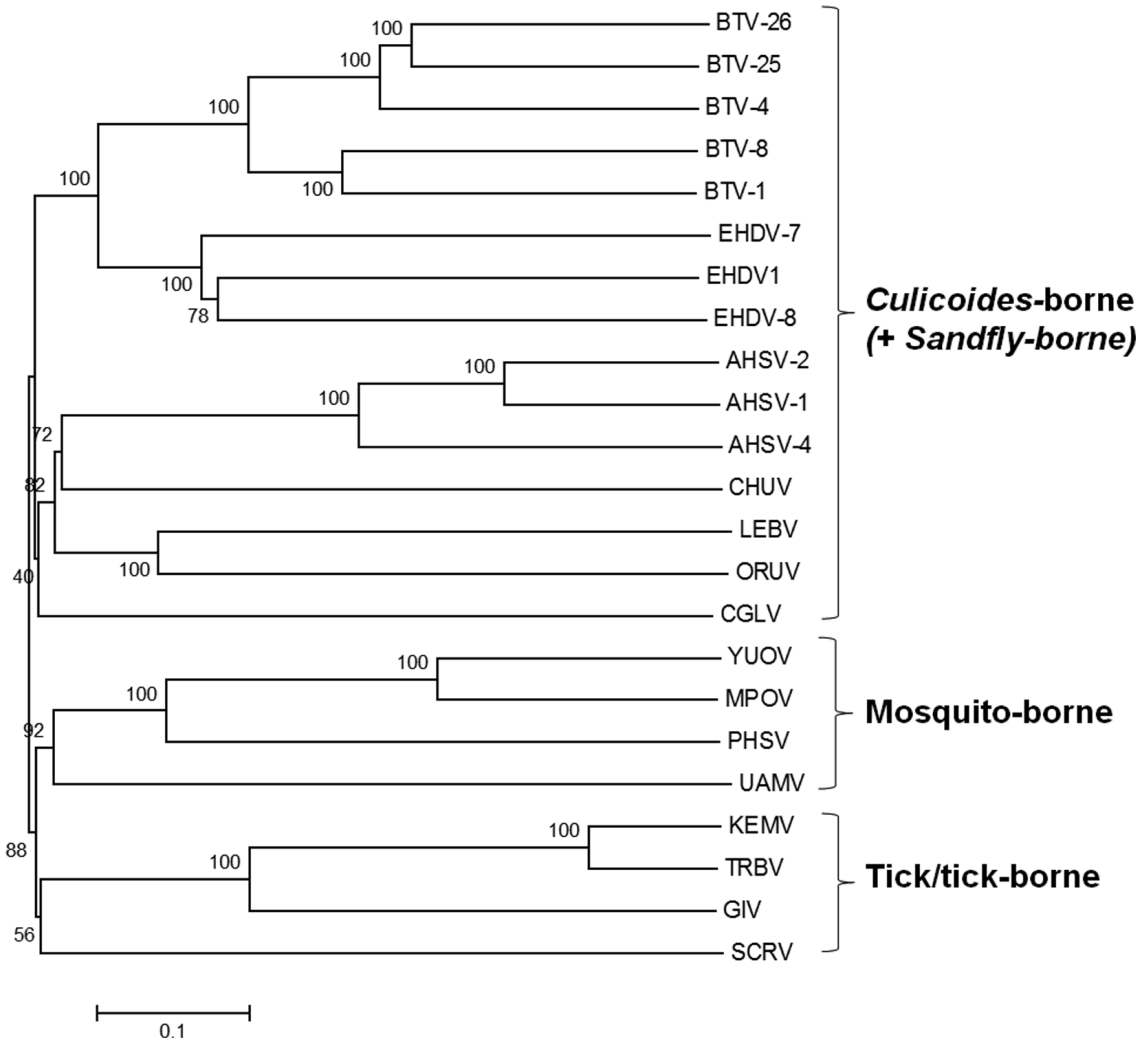
Neighbour-joining Amino acid tree depicting the three groups of tick-borne, mosquito-borne and *Culicoides*-borne OC1. The topology of orbivirus OC1-based tree is similar to that of the T2 protein and the VP7 coding nucleotide sequence-based trees. LEBV, ORUV and CGLV all cluster among *Culicoides*-borne orbiviruses. The scale bar represents the number of substitutions per site.

**Figure 11:**
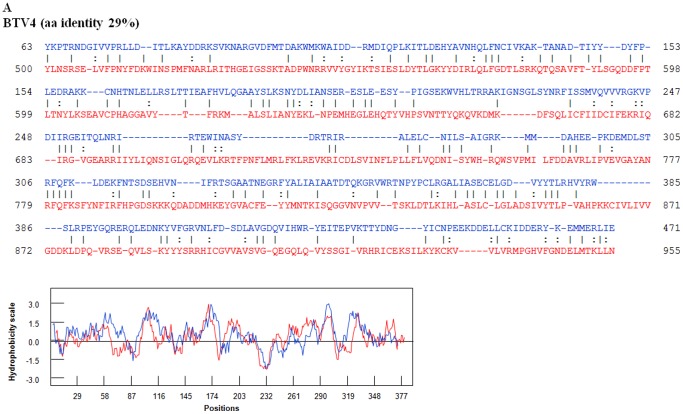
Potential duplications in BTV VP2(OC1). OC1 sequence of BTV VP2 with repeats identified by REPRO. The sequences in blue font represent the NH2 terminal domain while the sequences in the red font represent the COOH terminal domain. Superimposed hydrophobicity profiles of the two domains are shown below each alignment. The amino acid identity between the two identified repeats is 29%. Hydrophobicity profiles of repeats are shown below the alignment. In BTV VP2, aa 63 to 471 were identified as a repeat of aa 500 to 955. Finer sequence analyses identified that aa75–442 have highly similar hydrophobicity plots to aa 567–955.

**Figure 12:**
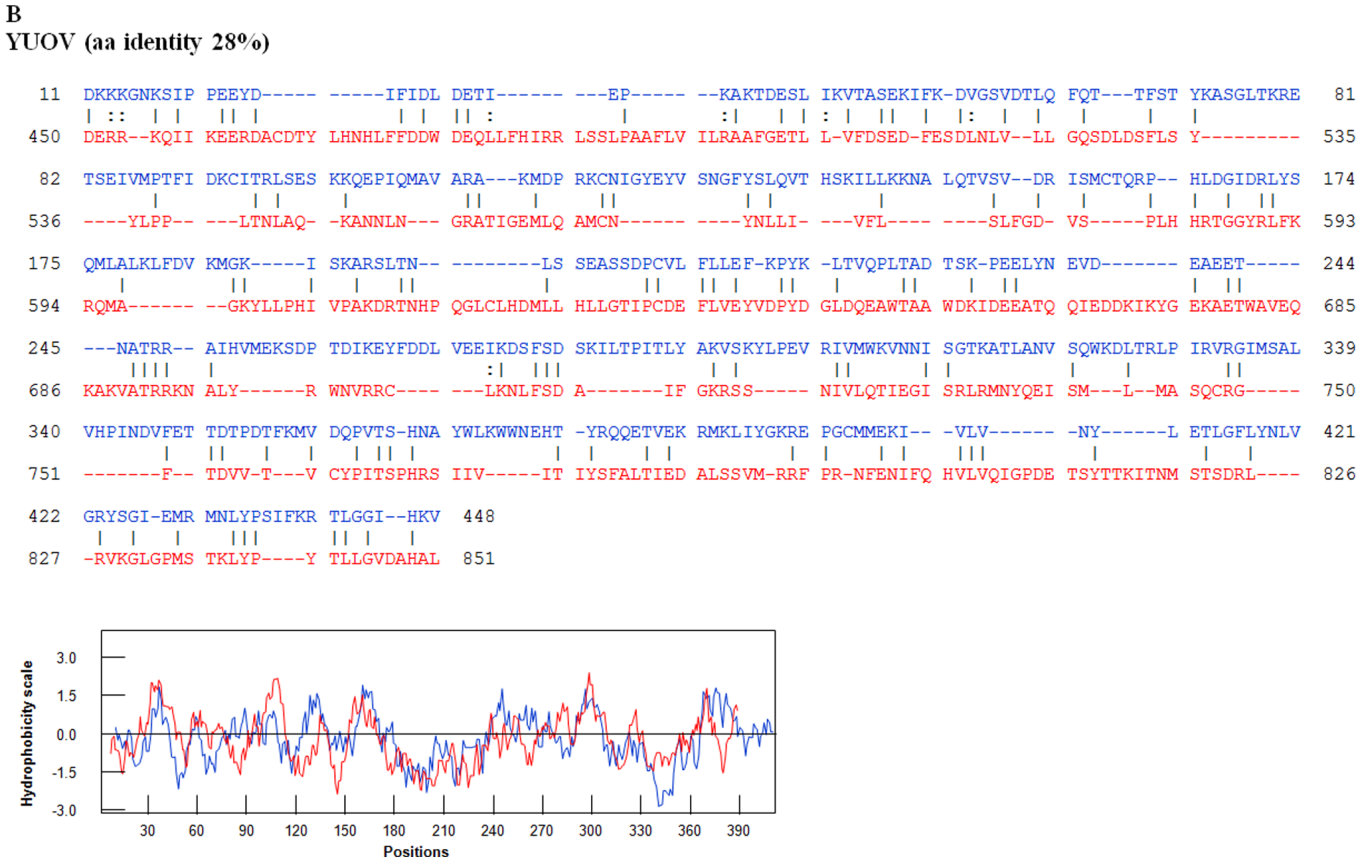
Potential duplications in YUOV VP3(OC1). OC1 sequence of YUOV VP3 with repeats identified by REPRO. The sequences in blue font represent the NH2 terminal domain while the sequences in the red font represent the COOH terminal domain. Superimposed hydrophobicity profiles of the two domains are shown below each alignment. The amino acid identity between the two identified repeats is 28%. Hydrophobicity profiles of repeats are shown below the alignment. In YUOV VP3, aa 11 to 448 were identified as a repeat of aa 45 to 851. Finer sequence analysis identified that aa 60–448 have highly similar hydrophobicity plots to aa 462–851.

An amino acid based neighbour joining phylogenetic tree shows three groups of the highly variable cell-attachment and outer-capsid protein ‘OC1’ (figure 10). Use of the programme ‘REPRO’ indicates that OC1 of the insect-borne viruses contains sequences that have been duplicated at some point during their evolution (figures 11 and 12). In BTV, aa 63 to 471 were identified as a repeat of aa 500 to 955. Finer sequence analyses identified that aa75–442 have highly similar hydrophobicity plots to aa 567–955 (figure 11). In YUOV, aa 11 to 448 were identified as a repeat of aa 45 to 851. Finer sequence analysis identified that aa 60–448 have highly similar hydrophobicity plots to aa 462–851 (figure 11).

**Figure 13:**
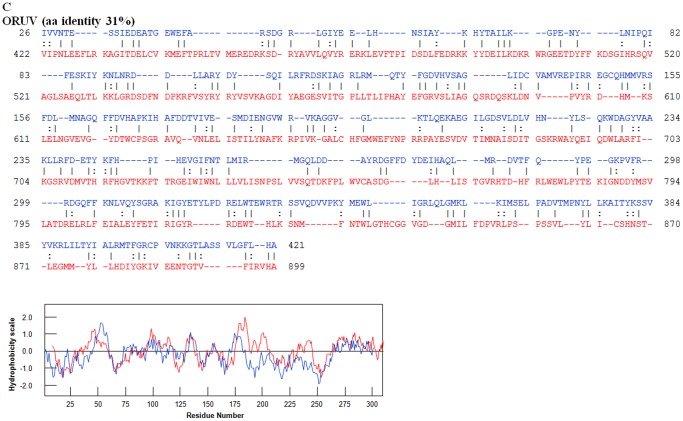
Potential duplications in ORUV VP2(OC1). OC1 sequence of ORUV VP2 with repeats identified by REPRO. The sequences in blue font represent the NH2 terminal domain while the sequences in the red font represent the COOH terminal domain. Superimposed hydrophobicity profiles of the two domains are shown below each alignment. The amino acid identity between the two identified repeats is 31%. Hydrophobicity profiles of repeats are shown below the alignment. In ORUV VP2, aa 26 to 421 were identified as a repeat of aa 427 and 899 of the same protein. Finer sequence analysis identified that aa 75–384 have highly similar hydrophobicity plots to aa 520–899.

**Figure 14:**
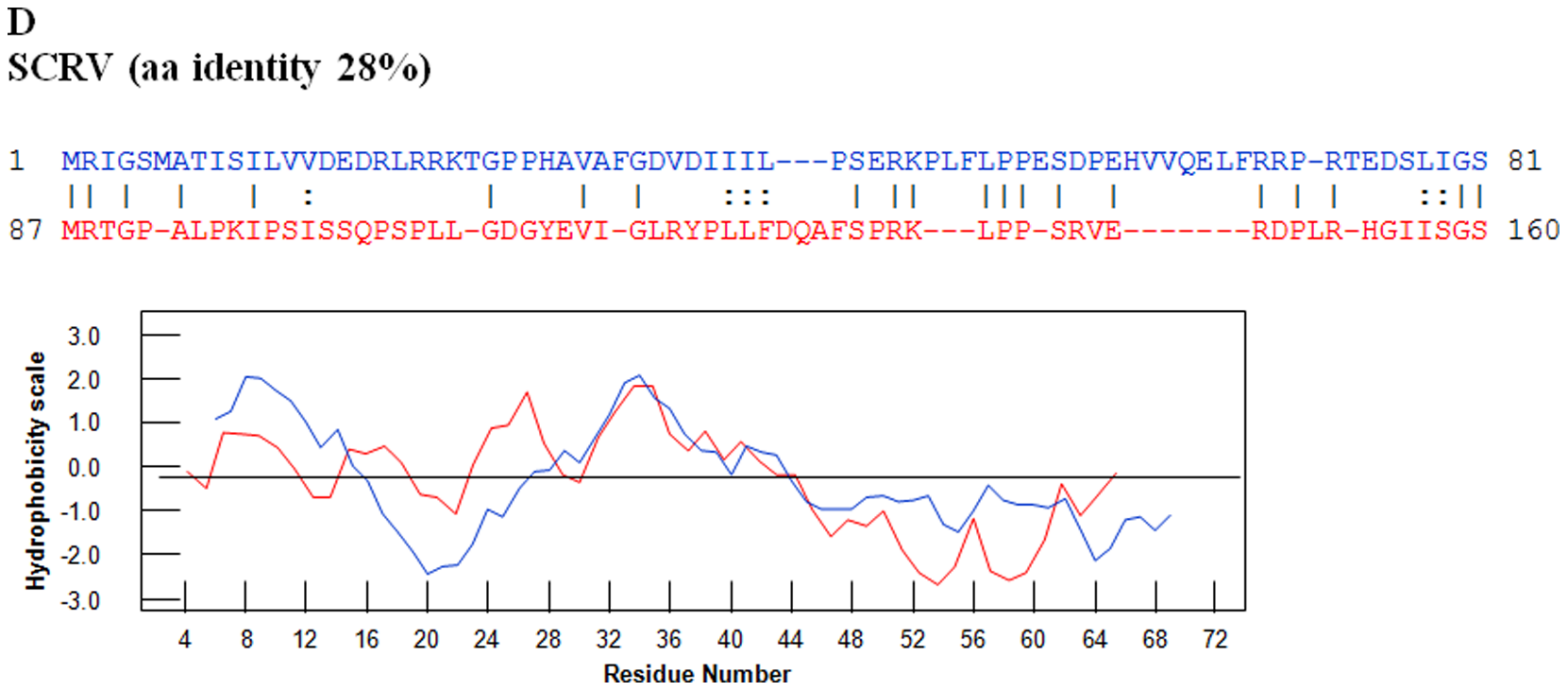
Potential duplications in SCRV VP3(OC1). OC1 sequence of SCRV VP3 with repeats identified by REPRO. The sequences in blue font represent the NH2 terminal domain while the sequences in the red font represent the COOH terminal domain. Superimposed hydrophobicity profiles of the two domains are shown below each alignment. The amino acid identity between the two identified repeats is 28%. In SCRV VP3, aa 1 to 81 of SCRV may also represent a duplication of aa 88 to 160. Hydrophobicity profiles of repeats are shown below the alignment. The hydrophobicity plots of the two sequences in SCRV VP3 are similar.

For the three viruses characterised in this study, OC1 is identified as VP2 (based on its relative size, as the second largest virus-protein). In ORUV (figure 13), aa 26 to 421 of VP2(OC1) was identified as a repeat of aa 427 and 899 of the same protein. Finer sequence analysis identified that aa 75–384 have highly similar hydrophobicity plots to aa 520–899 (figure 13). In LEBV, aa 7 to 412 of VP2(OC1) represents a repeat of aa 417 to 831. In CGLV, which has the longest orbivirus OC1 reported to date (1151 aa), aa 1 to 505 were derived by duplication of aa 521 to 1002. In the tick-borne orbiviruses, OC1 of SCRV is also identified as VP3, containing 654 aa, while OC1 of tick borne KEMV and GIV is identified as VP4, containing 551 aa. Amino acids 1 to 81 of SCRV VP3(OC1) may also represent a duplication of aa 88 to 160 (figure 14). The hydrophobicity plots of the two sequences are highly similar (figure 14).

## Discussion

The full-genome sequences of three zoonotic orbiviruses (ORUV, LEBV and CGLV) are reported here. ORUV infects primates (including humans), camels, bovines, caprines and ovines. LEBV infects humans and rodents, while CGLV infects humans, rodents and sloths [5], [7], [8], [10], [11], [12], [42], [43], [44] (http://wwwn.cdc.gov/arbocat). The sequences, of ORUV, LEBV and CGLV, were used to analyse their relationships to other previously characterised orbiviruses.

Within the genus *Orbivirus*, the most conserved proteins are VP1(Pol), T2 and T13. The VP7(T13) protein which forms the outer layer of the virus-core, can mediate cell attachment and infection by core particles, is immuno-dominant and represents a primary antigenic determinant of virus serogroup (virus species) [1], [2], [45]. VP7(T13) of ORUV, LEBV and CGLV shows highest identity levels with the *Culicoides*-borne viruses (e.g. 40–52% with BTV), but much lower levels with those of the tick-borne viruses (e.g. 21–25% with GIV) or the mosquito-borne viruses (e.g. 22–23% with YUOV).

The orbivirus sub-core shell T2 protein plays a major functional role in virus protein/RNA structure and particle-assembly [31], [46] exhibiting very high levels of sequence identity (>91%) within a single *Orbivirus* species (serogroup). The T2 proteins of ORUV, LEBV and CGLV also show closer relationships with the *Culicoides*-borne viruses (e.g. 56–68% with BTV), but again much lower identity with tick-borne (e.g. 36–37% with GIV) and mosquito-borne orbiviruses (e.g. 22–23% with YUOV). The same situation is found in VP1(Pol) (55–61% with BTV, 45% with YUOV, 44–47% with GIV). These aa identities are consistent with phylogenetic groupings of ORUV, LEBV and CGLV with the *Culicoides*-borne orbiviruses.

Single isolates of ORUV have been obtained from *Culex perfuscus*, *Anopheles gambiae*, or *Aedes aegypti* mosquitoes) [3], [7] (http://wwwn.cdc.gov/arbocat). The inability of orally fed *Aedes aegypti* to transmit ORUV, even though transmission occurred after intra-thoracic inoculation (bypassing a potential gut barrier), raises questions about the current assumption that this virus is mosquito-borne.

Differences in the migration order of the T2 protein, between the groups of orbiviruses that are transmitted by different vectors, are caused by large variations in the relative size of the highly variable outer-capsid protein one (OC1). In the *Culicoides*-borne orbiviruses, OC1 is the 2^nd^ largest viral protein (VP2 - encoded by Seg-2: 110–120 kDa), while in the mosquito-borne viruses it is slightly (∼10%) smaller (VP3 - encoded by Seg-3: 90–100 kDa).

Our analyses indicate that OC1 of both groups of insect-borne orbiviruses were generated by concatermerisation/duplication of OC1, from an ancestral tick borne virus. The ancestral form of OC1 would be ∼573 aa, similar in length to that Great Island virus (GIV) which is transmitted by ticks, contains a smaller OC1 (VP4 - encoded by Seg-5, 62 kDa) that is approximately ∼55% of the size of its counterpart in the insect-borne orbiviruses [13]. The OC1 and T2 proteins of ORUV, LEBV and CGLV are identified as VP2 and VP3 respectively, again grouping them with the *Culicoides*-borne viruses.

Phylogenetic analysis of tick-, mosquito- and *Culicoides*-borne sequences indicate that OC1 of the tick-borne viruses forms a distinct phylogenetic cluster, which has a common root with OC1 of the mosquito-borne orbiviruses, while the *Culicoides*-borne OC1 forms a further separate cluster. These analyses also indicate two groups of OC1 representing the tick or tick-borne orbiviruses. One represented by VP4(OC1) of the GIV group, which may be closer to the ancestral orbivirus, while the other group is represented by SCRV VP3(OC1) which has a partial duplication within its first 160 aa.

Duplication of individual orbivirus genome segments can occur via a process of concatermerisation [47]. Partial or full gene duplication (concatermerisation) has also been identified in several other ‘reovirus’ proteins, indicating that it is a generalised mechanism creating sequence diversity in viruses of family *Reoviridae* [47], [48], [49], [50]. For example the genome segment 9 of Colorado tick-fever virus (*Coltivirus*, *Reoviridae*) was found to be generated by a full gene duplication. Following a duplication-event the repeated sequences can evolve separately, in response to functional constraints [48], [49], [51].

The two subdomains of the insect-borne OC1s show significant levels of aa identity (28–29%) and have very similar (almost superimposable) hydrophobicity profiles. It therefore appears unlikely that the smaller OC1s of the tick-borne orbiviruses could have originally evolved through partial deletion of a larger (insect-borne) precursor protein. Deletions in dsRNA viruses often generate defective-interfering viruses, that are unable to spread in the absence of the original complementing virus-strain [50], [52], while concatermerisation does not affect the viability of the viruses [47], [53]. Recently, an African horse sickness virus expressing a truncated VP2 from which 20% of the protein (amino acids 279 to 503) had been lost, was found to replicate efficiently in cell culture [54]. In BTV VP2, the corresponding sequence to this deletion encompasses the neutralisation epitopes described earlier [55]. The size differences observed in OC1 between the different groups of orbiviruses may have implications for their interactions with cell-surface receptors in the different groups of vectors/hosts. Antibody-neutralisation of the tick-borne orbiviruses involves both OC1 and OC2, while OC1 is clearly the dominant neutralisation antigen of the insect-borne orbiviruses [1], [2], [56]. From an evolutionary perspective, for such a duplication of aa sequence to become fixed within the virus population it must provide a fitness-advantage in terms of replication or transmission efficiency, promoting survival of the new modified gene, for example through adaptation to new environment/host [57], [58], [59]. Mutations that positively affect protein function could potentially increase, rather than reduce the probability of retention of the duplicated gene [60]. After a concatermeric duplication of the aa sequence within a single protein, subsequent evolution of the duplicated gene could lead to partitioning and separation of its original functions into the different halves of the protein, rather than simply a duplication of these functions [60]. Indeed, this may be true for OC1 of the insect-borne orbiviruses such as BTV, where neutralisation epitopes are principally mapped to the amino half of VP2(OC1) [55], [61]. It is noteworthy that the deletion that was identified in AHSV VP2 implicates a sequence of domain 1, while domain 2 is intact. It has been suggested that this deletion also uncovers a sialic acid binding site [54], which may be located on domain 2. Domains 1 and 2 of BTV VP2(OC1) expressed separately, in a soluble form, were both found to raise neutralising antibodies in mice. However, the neutralising antibody titers were 10 times higher with domain 1 than domain 2 (Mohd Jaafar et al., manuscript in preparation).

The G+C content of ORUV, LEBV genomes also places them closer to the *Culicoides*-borne viruses, than to the mosquito-borne viruses, while the G+C content of CGLV (transmitted by sandflies) is borderline between those of mosquito-borne and *Culicoides*-borne viruses.

Based on their isolation from mosquitoes, both ORUV and LEBV were originally considered likely to be mosquito-borne. However, it is possible for a virus to be isolated from freshly engorged mosquitoes that have ingested infectious blood meal, rather than an actual infection of the mosquitoes, which would be required for transmission. Although data presented here indicates that both ORUV, LEBV are likely to be *Culicoides*-borne viruses, this will require confirmation by vector competence studies. CGLV, the only known sandfly-borne orbivirus, clusters among the *Culicoides*-borne viruses. Interestingly, CGLV was also found to replicate in KC cells (cells derived from *Culicoides variipennis*) (data not shown).

It has been previously suggested that the non-vectored dsRNA viruses have evolved by co-evolution with their respective hosts [62]. Neighbour-joining analysis of orbivirus T2 proteins using the P-distance or Poisson’s correction algorithms, as well as maximum likelihood analyses, indicate that SCRV represents the oldest known orbivirus lineage, providing a ‘root’ for all of the other orbiviruses. SCRV has no known vertebrate hosts and could be a true “tick virus” rather than a “tick-borne virus” [13], [30], [63]. The same analyses also show that T2 proteins of the tick-borne and mosquito-borne orbiviruses form distinct phylogenetic clusters originating from a common branch, but are more closely related to each other than to the *Culicoides*-borne viruses, which are located on a distinct branch of the tree.

The VP7(T13)-based amino acid trees showed similar topologies to those of the T2 protein. Together with the amino acid or nucleic ML trees for Seg-1/VP1(Pol), these indicate that the tick-borne orbiviruses group together, providing a root for the mosquito-borne and *Culicoides*-borne orbivirus groups. Previous phylogenetic analyses, based on mitochondrial genes, indicate that ticks also represent a root for other arthropods, including the flies (*Culicoides* and sandflies) and mosquitoes [64]. The clustering of ORUV and LEBV among *Culicoides*-borne viruses disagrees with previous suggestions based on their isolation from mosquitoes, that both viruses are mosquito-borne.

A linear relationship was observed between the largest genetic distances calculated within each of the three phylogenetic groups of orbiviruses and dates for the evolutionary separation of their vectors. The similar topology of the viral-gene trees and vector-COXI based trees or antigen 5-related protein based trees is not shared with the mammalian-host-COXI tree (data not shown) and no linearity was detected between the genetic distances between viruses and the dates of separation of their mammalian hosts (data not shown). These results provide primary evidence for co-evolution of the orbiviruses with their arthropod vectors rather than their vertebrate hosts.

The G+C content of the mosquito genome is within the range of 35.2%–38.7% [65], [66] (http://www.broadinstitute.org/annotation/genome/aedes_aegypti.2/SingleGenomeIndex.html). In contrast the G+C content of ixodid tick genome is approximately 56% for coding regions (http://mail.vectorbase.org:82/pipermail/iscapularis/2008-December/000017.html). From available *Culicoides* sequences in the databases, the G+C content of a *Culicoides* coding region is approximately 39%. The G+C content of the genome of different vector-groups of orbiviruses is similar to those of their vectors, supporting the co-evolution hypothesis between orbiviruses and their respective hosts.

The G+C content is significantly different between the tick-borne and insect-borne orbiviruses (14% to 17% difference). This is inconsistent with a simple and rapid jump to a new vector species but suggests a much slower co-evolution/adaptation process. In contrast there are smaller differences in G+C content between the tick-borne and insect-borne flaviviruses (of only <9%) which appear to have diverged more recently from a proposed mosquito−/mosquito-borne ancestor [67], [68]. In phylogenetic trees, the insect-borne flaviviruses provide a ‘root’ for the tick-borne flaviviruses, while the reverse is true for the orbiviruses.

Previous evolutionary studies suggest that ticks appeared approximately 225 million years ago (MYA) [32], while the earliest dating of culicine mosquitoes is about 150 MYA [33]. *Culicoides* biting midges are vectors for several orbiviruses and their appearance has been dated to the Cretaceous period (140-65 MYA) [34], [35].

The topologies of phylogenetic trees for the orbivirus genes/proteins are similar to those of the vector’s genes. The relationship between genetic distances of the orbivirus genes and the dates of separation of the three vector groups (ticks, mosquitoes and midges) are near linear. OC1 of the insect-borne orbiviruses appears to have evolved from an ancestral OC1, probably from a tick-borne virus. It is therefore likely that orbiviruses have co-evolved with their vector groups generating three major phylogenetic groups. The available data suggest that viruses in these groups do not cross between the vector-species groups. The lack of co-speciation with their vertebrate hosts suggests that the ancestral orbiviruses were primarily arthropod viruses that subsequently crossed the species barrier between arthropods and mammalian hosts.

Based on the T2 gene (which showed the lowest rates of change in both the tick-borne and insect-borne orbiviruses), the most recent common ancestor of the known tick-borne orbiviruses is dated to ∼7,000 years ago (range: ∼4,500 to ∼8,500), while the most recent common ancestor for the currently known insect-borne orbiviruses is dated to 3,700 years ago (range: ∼2100 to ∼5200).

The data provided in this manuscript supports the co-evolution hypothesis for the orbiviruses with their vectors [13], indicating that it is more likely than host switching from one vector group to another. Isolates of a single virus species can be transmitted by more than one vector species (e.g. BTV has been isolated from several *Culicoides* species), making it difficult to infer co-speciation at the vector-species level. The earliest orbiviral ancestor was a tick/tick-borne orbivirus which existed at least 225 MYA. Mosquito or mosquito-borne orbiviral ancestors would have evolved from this ancestral virus followed by *Culicoides* or *Culicoides*-borne orbiviruses.

The generation of full genome sequence data for ORUV, LEBV and CGLV will facilitate the development of sequence-specific RT-PCR assays for epidemiological studies, well as identification of other virus isolates belonging to the same *Orbivirus* species.

## Supporting Information

Figure S1 A maximum likelihood tree showing phylogenetic comparisons of the nucleotide sequences of Seg-1 encoding the VP1(Pol) of ORUV, LEBV and CGLV, aligned with those of other *Orbivirus* species. The figure depicts the three groups of orbiviruses (i-*Culicoides-*/sandfly-borne, ii- mosquito-borne and iii- tick-borne) as separate clusters. The tree is based on codon to codon nucleotide alignments generated from aa profile alignment. The scale bar represents the number of substitutions per site.
(DOCX)

Figure S2 A maximum likelihood tree showing phylogenetic comparisons of the amino acid sequences of VP7(T13) protein of ORUV, LEBV and CGLV, aligned with those of other *Orbivirus* species. The figure shows a similar topology to that of the T2 proteins. The scale bar represents the number of substitutions per site.
(DOCX)

## References

[1] Attoui H, Mertens PPC, Becnel J, Belaganahalli M, Bergoin M, et al.. (2011) The Double Stranded RNA Viruses. In: King AMQ, Carstens EB, Lefkowitz EJ, editors. Virus Taxonomy: Ninth Report of the International Committee on Taxonomy of Viruses. London: Academic Press. 497–637.

[2] Mertens PPC, Attoui H, Duncan R, Dermody TS (2005) Reoviridae. In: Fauquet C, Mayo MA, Maniloff J, Desselberger U, Ball LA, editors. Virus Taxonomy Eighth Report of the International Committee on Taxonomy of Viruses. London: Elsevier/Academic Press. 447–454.

[3] Brown SE, Morrison HG, Karabatsos N, Knudson DL (1991) Genetic relatedness of two new Orbivirus serogroups: Orungo and Lebombo. J Gen Virol 72 (Pt 5): 1065–1072. 10.1099/0022-1317-72-5-1065 2033390

[4] Tomori O, Fabiyi A (1977) Orungo virus: a new agent from mosquitoes and man in Uganda and Nigeria. Nigerian Medical Journal 7: 5–8.

[5] Tomori O, Fabiyi A (1976) Neutralizing antibodies to Orungo virus in man and animals in Nigeria. Trop Geogr Med 28: 233–238. 827036

[6] Tomori O, Aitken TH (1978) Orungo virus: transmission studies with Aedes albopictus and Aedes aegypti (Diptera: Culicidae). J Med Entomol 14: 523–526. 633290 10.1093/jmedent/14.5.523

[7] CDC (2010) International Catalogue of Arboviruses Including Other Viruses of Vertebrates. 6/17/2010 ed. Fort Collins, Colorado: Centers for disease control and prevention.

[8] Tomori O, Fabiyi A, Murphy F (1976) Characterization of Orungo virus, an orbivirus from Uganda and Nigeria. Arch Virol 51: 285–298. 973798 10.1007/BF01317932

[9] Tomori O, Fabiyi A (1977) Susceptibility of laboratory and domestic animals to experimental infection with Orungo virus. Acta Virol 21: 133–138. 17281

[10] Moore DL, Causey OR, Carey DE, Reddy S, Cooke AR, et al (1975) Arthropod-borne viral infections of man in Nigeria, 1964–1970. Ann Trop Med Parasitol 69: 49–64. 1124969 10.1080/00034983.1975.11686983

[11] Travassos da Rosa AP, Tesh RB, Pinheiro FP, Travassos da Rosa JF, Peralta PH, et al (1984) Characterization of the Changuinola serogroup viruses (Reoviridae: Orbivirus). Intervirology 21: 38–49. 6698758 10.1159/000149501

[12] Seymour C, Peralta PH, Montgomery GG (1983) Viruses isolated from Panamanian sloths. Am J Trop Med Hyg 32: 1435–1444. 6316795 10.4269/ajtmh.1983.32.1435

[13] Belhouchet M, Mohd Jaafar F, Tesh R, Grimes J, Maan S, et al (2010) Complete sequence of Great Island virus and comparison with the T2 and outer-capsid proteins of Kemerovo, Lipovnik and Tribec viruses (genus Orbivirus, family Reoviridae). J Gen Virol 91: 2985–2993. 20739272 10.1099/vir.0.024760-0

[14] Attoui H, Billoir F, Cantaloube JF, Biagini P, de Micco P, et al (2000) Strategies for the sequence determination of viral dsRNA genomes. J Virol Methods 89: 147–158. 10996648 10.1016/s0166-0934(00)00212-3

[15] Attoui H, Billoir F, Biagini P, de Micco P, de Lamballerie X (2000) Complete sequence determination and genetic analysis of Banna virus and Kadipiro virus: proposal for assignment to a new genus (Seadornavirus) within the family Reoviridae. J Gen Virol 81: 1507–1515. 10811934 10.1099/0022-1317-81-6-1507

[16] Thompson JD, Gibson TJ, Plewniak F, Jeanmougin F, Higgins DG (1997) The CLUSTAL_X windows interface: flexible strategies for multiple sequence alignment aided by quality analysis tools. Nucleic Acids Res 25: 4876–4882. 9396791 10.1093/nar/25.24.4876 PMC147148

[17] Kumar S, Nei M, Dudley J, Tamura K (2008) MEGA: a biologist-centric software for evolutionary analysis of DNA and protein sequences. Brief Bioinform 9: 299–306. 18417537 10.1093/bib/bbn017 PMC2562624

[18] Saitou N, Nei M (1987) The neighbor-joining method: a new method for reconstructing phylogenetic trees. Mol Biol Evol 4: 406–425. 3447015 10.1093/oxfordjournals.molbev.a040454

[19] Posada D (2008) jModelTest: phylogenetic model averaging. Mol Biol Evol 25: 1253–1256. 18397919 10.1093/molbev/msn083

[20] Drummond AJ, Nicholls GK, Rodrigo AG, Solomon W (2002) Estimating mutation parameters, population history and genealogy simultaneously from temporally spaced sequence data. Genetics 161: 1307–1320. 12136032 10.1093/genetics/161.3.1307 PMC1462188

[21] Drummond A, Pybus OG, Rambaut A (2003) Inference of viral evolutionary rates from molecular sequences. Adv Parasitol 54: 331–358. 14711090 10.1016/s0065-308x(03)54008-8

[22] Drummond AJ, Ho SY, Phillips MJ, Rambaut A (2006) Relaxed phylogenetics and dating with confidence. PLoS Biol 4: e88. 16683862 10.1371/journal.pbio.0040088 PMC1395354

[23] Drummond AJ, Rambaut A, Shapiro B, Pybus OG (2005) Bayesian coalescent inference of past population dynamics from molecular sequences. Mol Biol Evol 22: 1185–1192. 15703244 10.1093/molbev/msi103

[24] Xia X, Xie Z (2001) DAMBE: software package for data analysis in molecular biology and evolution. J Hered 92: 371–373. 11535656 10.1093/jhered/92.4.371

[25] Kyte J, Doolittle RF (1982) A simple method for displaying the hydropathic character of a protein. J Mol Biol 157: 105–132. 7108955 10.1016/0022-2836(82)90515-0

[26] Hennig L (2001) WinPep 2.11: novel software for PC-based analyses of amino acid sequences. Prep Biochem Biotechnol 31: 201–207. 11426706

[27] Belhouchet M, Mohd Jaafar F, Firth AE, Grimes JM, Mertens PP, et al (2011) Detection of a fourth orbivirus non-structural protein. PLoS One 6: e25697. 22022432 10.1371/journal.pone.0025697 PMC3192121

[28] Ratinier M, Caporale M, Golder M, Franzoni G, Allan K, et al (2011) Identification and characterization of a novel non-structural protein of bluetongue virus. PLoS Pathog 7: e1002477. 22241985 10.1371/journal.ppat.1002477 PMC3248566

[29] Lange A, McLane LM, Mills RE, Devine SE, Corbett AH (2010) Expanding the definition of the classical bipartite nuclear localization signal. Traffic 11: 311–323. 20028483 10.1111/j.1600-0854.2009.01028.x PMC2886731

[30] Attoui H, Stirling JM, Munderloh UG, Billoir F, Brookes SM, et al (2001) Complete sequence characterization of the genome of the St Croix River virus, a new orbivirus isolated from cells of Ixodes scapularis. J Gen Virol 82: 795–804. 11257184 10.1099/0022-1317-82-4-795

[31] Grimes JM, Burroughs JN, Gouet P, Diprose JM, Malby R, et al (1998) The atomic structure of the bluetongue virus core. Nature 395: 470–478. 9774103 10.1038/26694

[32] Klompen JS, Black WC, Keirans JE, Oliver JH Jr (1996) Evolution of ticks. Annu Rev Entomol 41: 141–161. 8546444 10.1146/annurev.en.41.010196.001041

[33] Calvo E, Pham VM, Marinotti O, Andersen JF, Ribeiro JM (2009) The salivary gland transcriptome of the neotropical malaria vector Anopheles darlingi reveals accelerated evolution of genes relevant to hematophagy. BMC Genomics 10: 57. 19178717 10.1186/1471-2164-10-57 PMC2644710

[34] Grimaldi D, Engel MS (2005) Evolution of the insects. Cambridge: Cambridge University Press.

[35] Grogan WL, Szadziewski R (1988) A new biting midge from the upper Cretaceous (Cenomanian) Amaber of New Jersy (Diptera: Ceratopogonidae). J Paleontol 62: 808–812.

[36] Borkent A (2001) Leptoconops (Diptera, Ceratopogonidae), the earliest extant lineage of biting midge, discovered in 120–122 million-year-old Lebanese amber. American Museum novitates; no. 3328. American museum novitates: American museum of natural history. 1–11.

[37] Azar D, Nel A, Solignac M, Paicheler JC, Bouchet F (1999) New genera and species of psychodoid flies from the Lower Cretaceous amberof Lebanon Palaeontology. 42: 1101–1136.

[38] Azar D, Nel A (2003) Fossil psychodoid flies and their relation to parasitic diseases. Mem Inst Oswaldo Cruz 98 Suppl 135–37. 12687760 10.1590/s0074-02762003000900007

[39] Friedrich M, Tautz D (1997) Evolution and phylogeny of the Diptera: a molecular phylogenetic analysis using 28S rDNA sequences. Syst Biol 46: 674–698. 11975338 10.1093/sysbio/46.4.674

[40] Charrel RN, De Micco P, de Lamballerie X (1999) Phylogenetic analysis of GB viruses A and C: evidence for cospeciation between virus isolates and their primate hosts. J Gen Virol 80 (Pt 9): 2329–2335. 10.1099/0022-1317-80-9-2329 10501484

[41] Zanotto PM, Gould EA, Gao GF, Harvey PH, Holmes EC (1996) Population dynamics of flaviviruses revealed by molecular phylogenies. Proc Natl Acad Sci U S A 93: 548–553. 8570593 10.1073/pnas.93.2.548 PMC40088

[42] Tomori O, El-Bayoumi SM, Fabiyi A (1976) Virological and serological studies of a suspected yellow fever virus outbreak in Mabudi area of Benue Plateau State of Nigeria. Niger Med J 6: 135–143. 16296128

[43] Tomori O (1978) Response of Erythrocebus patas monkeys to experimental infection with the orbivirus Orungo virus. Trans R Soc Trop Med Hyg 72: 230–233. 97815 10.1016/0035-9203(78)90199-2

[44] Monath TP, Craven RB, Adjukiewicz A, Germain M, Francy DB, et al (1980) Yellow fever in the Gambia, 1978–1979: epidemiologic aspects with observations on the occurrence of orungo virus infections. Am J Trop Med Hyg 29: 912–928. 7435793 10.4269/ajtmh.1980.29.912

[45] Attoui H, Maan SS, Anthony SJ, Mertens PPC (2009) Bluetongue virus, other orbiviruses and other reoviruses: Their relationships and taxonomy. In: Mellor PS, Baylis M, Mertens PPC, editors. Bluetongue. London: Elsevier/Academic Press. 23–52.

[46] Gouet P, Diprose JM, Grimes JM, Malby R, Burroughs JN, et al (1999) The highly ordered double-stranded RNA genome of bluetongue virus revealed by crystallography. Cell 97: 481–490. 10338212 10.1016/s0092-8674(00)80758-8

[47] Anthony SJ, Darpel KE, Belaganahalli MN, Maan N, Nomikou K, et al (2011) RNA segment 9 exists as a duplex concatemer in an Australian strain of epizootic haemorrhagic disease virus (EHDV): Genetic analysis and evidence for the presence of concatemers as a normal feature of orbivirus replication. Virology 420: 164–171. 21968198 10.1016/j.virol.2011.09.009

[48] Mohd Jaafar F, Goodwin AE, Belhouchet M, Merry G, Fang Q, et al (2008) Complete characterisation of the American grass carp reovirus genome (genus Aquareovirus: family Reoviridae) reveals an evolutionary link between aquareoviruses and coltiviruses. Virology 373: 310–321. 18191982 10.1016/j.virol.2007.12.006

[49] Mohd Jaafar F, Attoui H, De Micco P, De Lamballerie X (2004) Termination and read-through proteins encoded by genome segment 9 of Colorado tick fever virus. J Gen Virol 85: 2237–2244. 15269364 10.1099/vir.0.80019-0

[50] Cao D, Barro M, Hoshino Y (2008) Porcine rotavirus bearing an aberrant gene stemming from an intergenic recombination of the NSP2 and NSP5 genes is defective and interfering. J Virol 82: 6073–6077. 18417592 10.1128/JVI.00121-08 PMC2395156

[51] Gibbs A, Keese PK (1995) In search of the origins of viral genes. In: Gibbs A, Calisher CH, Garcia-Arenal F, editors. In Molecular basis of virus evolution. New York/Melbourne: Cambridge University Press. 76–90.

[52] Esteban R, Wickner RB (1988) A deletion mutant of L-A double-stranded RNA replicates like M1 double-stranded RNA. J Virol 62: 1278–1285. 3279233 10.1128/jvi.62.4.1278-1285.1988 PMC253138

[53] Hundley F, Biryahwaho B, Gow M, Desselberger U (1985) Genome rearrangements of bovine rotavirus after serial passage at high multiplicity of infection. Virology 143: 88–103. 2998015 10.1016/0042-6822(85)90099-6

[54] Manole V, Laurinmaki P, Van Wyngaardt W, Potgieter CA, Wright IM, et al (2012) Structural insight into African horsesickness virus infection. J Virol 86: 7858–7866. 22593166 10.1128/JVI.00517-12 PMC3421665

[55] DeMaula CD, Bonneau KR, MacLachlan NJ (2000) Changes in the outer capsid proteins of bluetongue virus serotype ten that abrogate neutralization by monoclonal antibodies. Virus Res 67: 59–66. 10773319 10.1016/s0168-1702(00)00130-1

[56] Moss SR, Ayres CM, Nuttall PA (1987) Assignment of the genome segment coding for the neutralizing epitope(s) of orbiviruses in the Great Island subgroup (Kemerovo serogroup). Virology 157: 137–144. 2435054 10.1016/0042-6822(87)90322-9

[57] Lara-Ramirez EE, Segura-Cabrera A, Guo X, Yu G, Garcia-Perez CA, et al (2011) New implications on genomic adaptation derived from the Helicobacter pylori genome comparison. PLoS One 6: e17300. 21387011 10.1371/journal.pone.0017300 PMC3046158

[58] Bratlie MS, Johansen J, Sherman BT, da Huang W, Lempicki RA, et al (2010) Gene duplications in prokaryotes can be associated with environmental adaptation. BMC Genomics 11: 588. 20961426 10.1186/1471-2164-11-588 PMC3091735

[59] Valli A, Lopez-Moya JJ, Garcia JA (2007) Recombination and gene duplication in the evolutionary diversification of P1 proteins in the family Potyviridae. J Gen Virol 88: 1016–1028. 17325376 10.1099/vir.0.82402-0

[60] Force A, Lynch M, Pickett FB, Amores A, Yan YL, et al (1999) Preservation of duplicate genes by complementary, degenerative mutations. Genetics 151: 1531–1545. 10101175 10.1093/genetics/151.4.1531 PMC1460548

[61] DeMaula CD, Heidner HW, Rossitto PV, Pierce CM, MacLachlan NJ (1993) Neutralization determinants of United States bluetongue virus serotype ten. Virology 195: 292–296. 7686312 10.1006/viro.1993.1377

[62] Attoui H, Fang Q, Mohd Jaafar F, Cantaloube JF, Biagini P, et al (2002) Common evolutionary origin of aquareoviruses and orthoreoviruses revealed by genome characterization of Golden shiner reovirus, Grass carp reovirus, Striped bass reovirus and golden ide reovirus (genus Aquareovirus, family Reoviridae). J Gen Virol 83: 1941–1951. 12124458 10.1099/0022-1317-83-8-1941

[63] Nuttall PA (2009) Molecular characterization of tick-virus interactions. Front Biosci 14: 2466–2483. 10.2741/3390 19273212

[64] Wilson K, Cahill V, Ballment E, Benzie J (2000) The complete sequence of the mitochondrial genome of the crustacean Penaeus monodon: are malacostracan crustaceans more closely related to insects than to branchiopods? Mol Biol Evol 17: 863–874. 10833192 10.1093/oxfordjournals.molbev.a026366

[65] Gomez SM, Eiglmeier K, Segurens B, Dehoux P, Couloux A, et al (2005) Pilot Anopheles gambiae full-length cDNA study: sequencing and initial characterization of 35,575 clones. Genome Biol 6: R39. 15833126 10.1186/gb-2005-6-4-r39 PMC1088967

[66] Holt RA, Subramanian GM, Halpern A, Sutton GG, Charlab R, et al (2002) The genome sequence of the malaria mosquito Anopheles gambiae. Science 298: 129–149. 12364791 10.1126/science.1076181

[67] Gould EA, de Lamballerie X, Zanotto PM, Holmes EC (2003) Origins, evolution, and vector/host coadaptations within the genus Flavivirus. Adv Virus Res 59: 277–314. 14696332 10.1016/s0065-3527(03)59008-x

[68] Cook S, Holmes EC (2006) A multigene analysis of the phylogenetic relationships among the flaviviruses (Family: Flaviviridae) and the evolution of vector transmission. Arch Virol 151: 309–325. 16172840 10.1007/s00705-005-0626-6

