# Supplementary figures and images for: Full-Genome Characterisation of Orungo, Lebombo and Changuinola Viruses Provides Evidence for Co-Evolution of Orbiviruses with Their Arthropod Vectors
Source: PLoS One. 2014 Jan 24;9(1):e86392. doi: 10.1371/journal.pone.0086392 (PMC3901712; doi:10.1371/journal.pone.0086392)

**
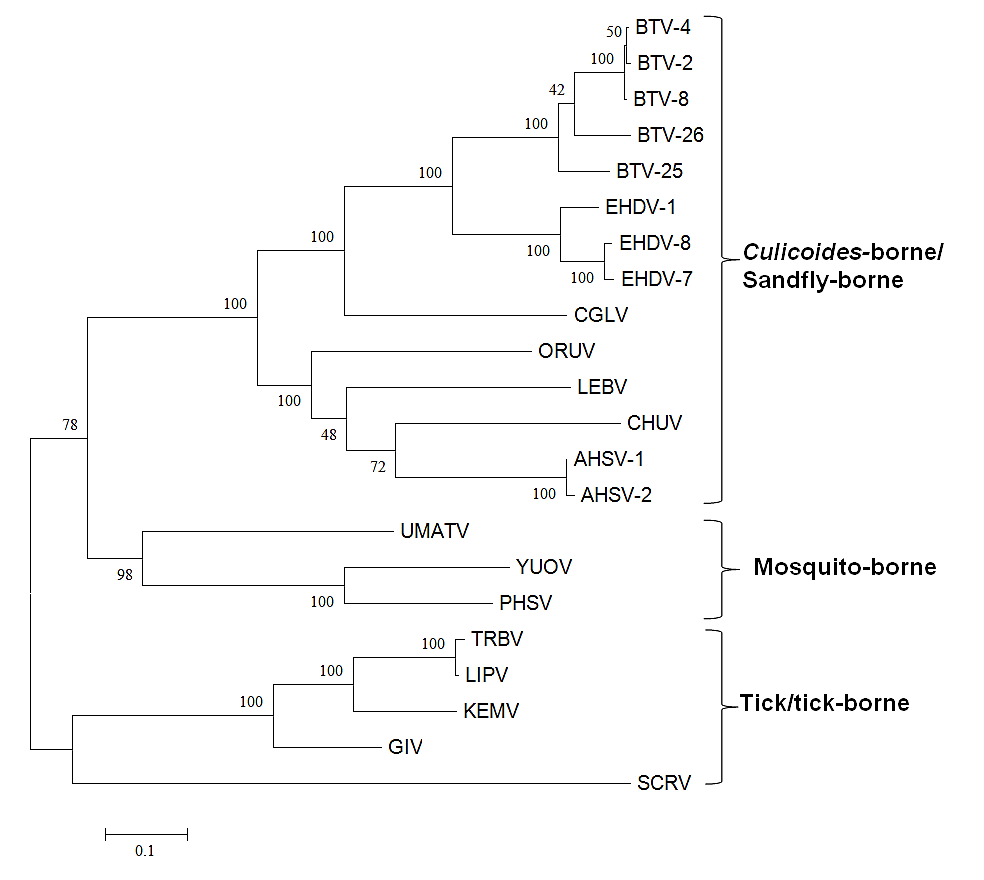
Figure S1**

Supplement: Figure S1 — A maximum likelihood tree showing phylogenetic comparisons of the nucleotide sequences of Seg-1 encoding the VP1(Pol) of ORUV, LEBV and CGLV, aligned with those of other Orbivirus species. The figure depicts the three groups of orbiviruses (i-Culicoides-/sandfly-borne, ii- mosquito-borne and iii- tick-borne) as separate clusters. The tree is based on codon to codon nucleotide alignments generated from aa profile alignment. The scale bar represents the number of substitutions per site. (DOCX) [file pone.0086392.s001.docx]

**Figure S2**


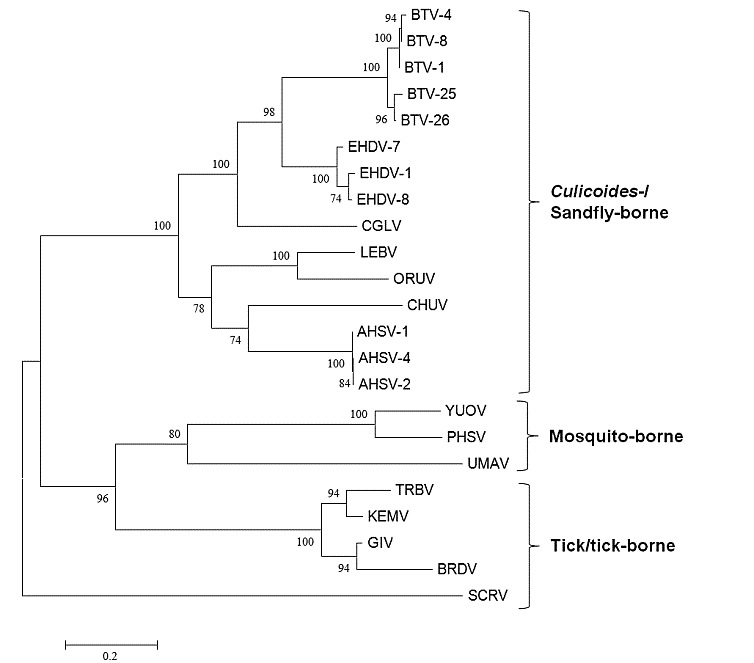

Supplement: Figure S2 — A maximum likelihood tree showing phylogenetic comparisons of the amino acid sequences of VP7(T13) protein of ORUV, LEBV and CGLV, aligned with those of other Orbivirus species. The figure shows a similar topology to that of the T2 proteins. The scale bar represents the number of substitutions per site. (DOCX) [file pone.0086392.s002.docx]
